# Does the Neighborhood Area of Residence Influence Non-Attendance in an Urban Mammography Screening Program? A Multilevel Study in a Swedish City

**DOI:** 10.1371/journal.pone.0140244

**Published:** 2015-10-13

**Authors:** Magdalena Lagerlund, Juan Merlo, Raquel Pérez Vicente, Sophia Zackrisson

**Affiliations:** 1 Department of Translational Medicine, Diagnostic Radiology, Lund University, Skåne University Hospital, Malmö, Sweden; 2 Unit for Social Epidemiology, Department of Clinical Sciences, Faculty of Medicine, Lund University, Malmö, Sweden; Örebro University, SWEDEN

## Abstract

**Background and aim:**

The public health impact of population-based mammography screening programs depends on high participation rates. Thus, monitoring participation rates, as well as understanding and considering the factors influencing attendance, is important. With the goal to acquire information on the appropriate level of intervention for increasing screening participation our study aimed to (1) examine whether, over and above individual factors, the neighborhood of residence influences a woman’s mammography non-attendance, and (2) evaluate, whether knowing a woman’s neighborhood of residence would be sufficient to predict non-attendance.

**Methods:**

We analyze all women invited to mammography screening in 2005–09, residing in the city of Malmö, Sweden. Information regarding mammography screening attendance was linked to data on area of residence, demographic and socioeconomic characteristics available from Statistics Sweden. The influence of individual and neighborhood factors was assessed by multilevel logistic regression analysis with 29,901 women nested within 212 neighborhoods.

**Results:**

The prevalence of non-attendance among women was 18.3%. After adjusting for individual characteristics, the prevalence in the 212 neighborhoods was 3.6%. Neighborhood of residence had little influence on non-attendance. The multilevel analysis indicates that 8.4% of the total individual differences in the propensity of non-attendance were at the neighborhood level. However, when adjusting for specific individual characteristics this general contextual effect decreased to 1.8%. This minor effect was explained by the sociodemographic characteristic of the neighborhoods. The discriminatory accuracy of classifying women according to their non-attendance was 0.747 when considering only individual level variables, and 0.760 after including neighborhood level as a random effect.

**Conclusion:**

Our results suggest that neighborhoods of residence in Malmö, Sweden (as defined by small-area market statistics (SAMS) areas) do not condition women’s participation in population based mammography screening. Thus, interventions should be directed to the whole city and target women with a higher risk of non-attendance.

## Introduction

After a gradual introduction throughout Sweden, and following recommendations from the National Board of Health and Welfare [1], a nationwide program for population-based outreach mammography screening for women over the age of 40 or 50 was implemented by 1997 [2]. Non-attendance rates across Sweden have ranged between 9 and 34%, and have generally been lower in metropolitan regions [2, 3]. With the initial non-attendance rate of 35% during the period 1990–93 [4], Malmö had one of the highest non-attendance rates in Sweden. Despite controversies in the research community regarding the efficiency of mammography screening [5] the public health impact of population-based mammography screening programs still depends on high participation rates. Thus, monitoring participation rates, as well as understanding and considering the factors influencing non-attendance, is important.

Access on equal terms and according to needs is at the core of Swedish health care [6], but even in a context where mammography screening is invitational and free of charge or offered at a low out-of-pocket cost there may be other obstacles impeding accessibility. Associations between personal characteristics (e.g. demographic, socioeconomic and life-style factors, health status, history of breast disease, and knowledge and beliefs about breast cancer) and mammography attendance have been extensively examined, which is well demonstrated by a review of 195 studies of U.S. populations [7]. Besides, over and above individual level factors, a number of previous studies in Europe [8–11] Japan [12], and North America [13–19] have investigated geographical area of residence as a potential determinant of mammography attendance. Those studies have applied multilevel regression analyses [20–23] and mainly focused on the analyses of associations between area-level specific contextual variables (characteristics of the area such as socioeconomic deprivation, degree of urbanization, breast cancer incidence and mortality, density of mammography facilities; type of screening program such as population-based/opportunistic; and other health-care/institutional factors) and individual level mammography attendance. However, the analysis of *specific contextual influences* is based on differences between neighborhood averages and disregard individual heterogeneity of responses around neighborhood averages. As it has been explained elsewhere [22, 24–26] the exclusive study of measures of association provides insufficient information for understanding neighborhood influences. For this purpose, we also need to evaluate to which extend the neighborhood by itself condition individual level variance without specifying any neighborhood characteristic other than the very neighborhood boundaries used in the analysis. In multilevel analysis this *general contextual influence* is expressed by measures of variance such as the intra-class correlation coefficient [22]. Using this approach, a previous study of ours conducted in Malmö reported that the intra-neighborhood correlation for non-attendance in the mammography screening program during 1990–93 was only 4.3% and this small general contextual effect of neighborhood decreased substantially when adjusting for individual sociodemographic factors and neighborhood characteristics [4].

This distinction between *specific* and *general* contextual influences is fundamental [25] since in analogy with diagnostic, prognostic, or screening markers [27], measures of association provide limited information for gauging the relevance of the neighborhood on individual outcomes in general and on mammography attendance in particular. In fact, based on this analogy, it is possible to apply common measures of discriminatory accuracy as the area under the ROC curve (AU-ROC) for assessing neighborhood influences (see S1 File) [28, 29]. The AU-ROC measure is well recognized among clinicians and epidemiologist and facilitates an improved understanding when it comes to evaluating neighborhood effects on individual mammography non-attendance.

Our study had two main objectives. One objective was to examine whether, over and above individual factors, the neighborhood of residence influences a woman’s mammography non-attendance. The other objective was to evaluate, from a more pragmatic point of view, whether knowing a woman’s neighborhood of residence would be sufficient to predict non-attendance. Our goal is to acquire information on the appropriate level of intervention for increasing screening participation. When doing so we also apply an innovative analytical approach based on AU-ROC analysis that has been recently described by us (see S1 File) [28, 29]. We analyze all women in the city of Malmö that received an invitation to attend mammography screening during the period 2005 to 2009.

## Materials and Methods

### Study population

The Malmö Mammographic Screening Program is a population-based program that was established in Malmö, Sweden, in 1990. Malmö is located in the county of Scania, in southern Sweden, and the municipality had a population of 318,000 in 2014 [30]. There is one mammography screening unit to which female residents in the eligible age-range are invited by mail to attend screening for a small out-of-pocket-fee at intervals of 1.5–2 years, depending on their date of birth and breast density. The screening database is continuously updated with the population register. Because of changes in national recommendations, the age-group eligibility has varied somewhat over the years. Between 1990 and 1998, women in the age range 50–69 years were invited. In 1999, the upper age limit was extended to 74 years, in 2007 the lower age limit was decreased to 48 years, and in 2009 it was further lowered to 40 years.

For the purpose of this study we identified women who had been invited to Malmö Mammographic Screening Program between the years 2005 and 2009 (n = 37,311). A unique personal identification number is assigned to every resident in Sweden. This number permits accurately linking records between different population registers. The Swedish authorities (Statistics Sweden) replaced this number with an arbitrary code in order to protect the anonymity of the individuals in the study. Using this code, we linked the data from the Mammography Screening Register with several registers administered by the National Board of Health and Welfare (e.g., the Swedish Cancer Register and the Birth register) and by Statistics Sweden (e.g., the Register of Total Population and the Longitudinal Integration Database for Health Insurance and Labor Market Studies (LISA)). From these registers we obtained information on individual demographic and socioeconomic characteristics, migration, breast cancer diagnoses, and neighborhood area of residence.

**Fig 1:**
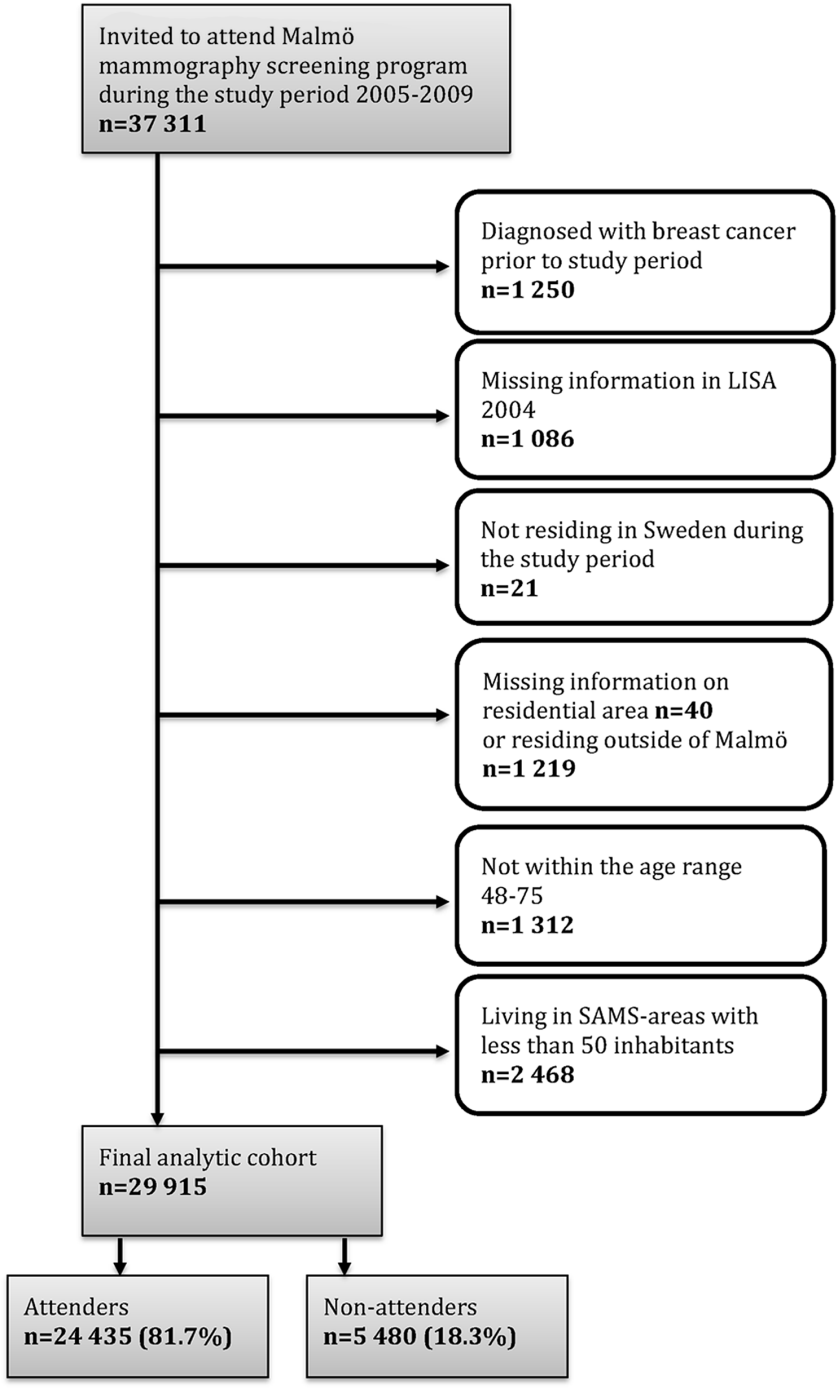
Selection of analytic cohort with stepwise exclusions.

From the 37,311 women in the mammography register we excluded women who had been diagnosed with breast cancer before the study period, who were missing in LISA, who were not residing in Malmö during the study period, who did not have a residential area code, and who were not in the age group 48–75. The intent of selecting this age range was to include an interval where women had been invited throughout most of the study period and to allow for a certain overflow from the age limit of 74 years, due to administrative reasons, e.g. rescheduling. We also excluded women who lived in smaller neighborhoods (<50 inhabitants), in order to insure a more robust sample size in each neighborhood. The sample flow chart in Fig 1 describes the different steps of exclusion, resulting in a final sample of 29,915 individuals (i.e. 80% of the original database)

### Assessment of individual-level variables

The outcome variable in our study was non-attendance in mammography screening (yes/no), according to the most recent screening invitation for each woman during the period 2005 to 2009. Other screening program related variables that were obtained from the Malmö Mammography Screening Register were year of invitation (2005, 2006, 2007, 2008 and 2009), season (Fall (Sept-Nov), Winter (Dec-Feb), Spring (March-May), Summer (June-Aug)) and number of screening invitations measured as a continuous variable (range: 1–15). Sociodemographic variables and their categorizations included age (48–50, 51–55… to 71–75) derived from year of birth, marital status (married, divorced, widowed and never married), having children living at home (yes/no), number of years of education (≤9 years, 10–12, >12), individual income as equalized household disposable income (quartiles), being employed (yes/no), country of birth (Sweden, Nordic country, Europe, and other), country of citizenship (Sweden, Nordic, European, and other), having migrated across the Swedish boarder more than once (yes/no), and having lived in Sweden for less than five years (yes/no). Missing values were included as a category for two of the variables (education and time in Sweden) in order to retain as many individuals in the analyses as possible. Individuals with missing values for country of birth (n = 2) and citizenship (n = 12) were excluded from the multilevel analysis. No other variables had missing values.

We assumed that the individual level variables could be confounders of the neighborhood differences. For instance, if age influences participation, neighborhood differences in non-attendance might be due to the different age composition of the neighborhoods rather to real contextual effects. In order to adjust for this possible compositional confounding we created a risk score (RS) representing the predicted probability of mammography non-attendance obtained from a multiple logistic regression modeling the individual variables indicated above (both program related and sociodemographic). The coefficients used for the risk score are presented in S1 Table. We categorized this risk score in 10 groups by deciles using the lowest decile (lowest probability of non-attendance) as the reference group in the analyses. The purpose of the RS was to perform a parsimonious adjustment for confounders rather than to create a prediction model valid for other contexts outside the database [31].

### Assessment of area-level variables

We defined neighborhoods on the basis of small-area market statistics (SAMS) from Statistics Sweden. SAMS refers to the smallest administrative area units in Sweden, and the SAMS boundaries are drawn to include similar types of housing in a neighborhood. We recorded an individual’s SAMS the year of her most recent screening invitation during the study period 2005 to 2009. A total of 335 neighborhoods were originally included in the database. In our analyses we only included 212 neighborhoods containing at least 50 women, which excluded 2,468 women.

A neighborhood sociodemographic index was created using a number of sociodemographic variables aggregated at the neighborhood level. This information was obtained from the Longitudinal Multilevel Analysis in Scania (LOMAS) database, containing all people living in Scania in the year 2005. The variables used included proportions of people who had low education (≤9 years), had low household income (lowest quartile: <92300 SEK), were not employed, were not born in Sweden, and had not lived in the same area during the period 2001–2005. To calculate these proportions we included both male and female residents in the age group 18–65 for education, income and employment, and all residents for the variables country of birth and mobility in and out of the area. The variables were firstly standardized (by subtracting the overall mean value to the specific value of the neighborhood and dividing by the standard deviation) in the population of 212 neighborhoods. Thereafter all variables were summed up to generate the neighborhood sociodemographic index. Finally, we categorized the neighborhoods according to the contextual index variable in deciles using the lowest decile score as the reference group in the analyses.

### Statistical analysis

The data presented a hierarchical structure consisting of women (first level) nested within neighborhoods (second level). Thus, we performed multilevel logistic regression analyses to estimate the probability of non-attendance at mammography screening [20–23, 32, 33]. The multilevel regression analysis accounted for the possible intra-neighborhood correlation of the individual level information. Accounting for this correlation is necessary in order to obtain correct statistical estimations of uncertainty (i.e. standard errors). Notably, the intra-neighborhood correlation is a variance partition coefficient that indicates the share of the total individual variance that is at the neighborhood level. The size of this coefficient is fundamental information in our study since the higher the coefficient is, the more relevant the neighborhood level is for understanding a woman’s non-attendance in mammography screening [22].

A main objective of our study was to find out if women’s neighborhood of residence influenced non-attendance in mammography screening over and above individual characteristics. That is, would a specific woman have had a different propensity for non-attendance if she were residing in a different neighborhood? How large is this influence? For this purpose we first performed a *single* level logistic regression (Model 1a) adjusting for individual level characteristics by means of the RS and calculated the AU-ROC. This model will also inform on the accuracy of the individual level information for predicting non-attendance in mammography screening without considering at all the neighborhood level.

Thereafter, we performed a *multilevel* logistic regression (Model 2) combining the RS and the random intercept for the neighborhood. A substantial improvement of the AU-ROC comparing with Model 1a would indicate that the neighborhood conditions women’s behavior and provides relevant information for predicting non-attendance in mammography screening in addition to knowing the individual RS and, therefore, interventions to improve mammography screening could be targeted to specific neighborhoods.

In the last model (Model 3) we added the neighborhood sociodemographic index. By doing so we would be able to know if a possible general neighborhood effect identified in Model 2 was mediated by the sociodemographic characteristics of the neighborhood.

Another, more pragmatic objective of our study was to examine how well we can predict non-attendance by only using the information of neighborhood of residence. In practice it is easier to obtain information on which neighborhood a woman resides in, than on her individual characteristics. For this purpose we performed a multilevel model (Model 1b), which included only a random intercept for the neighborhood. Model 1b will inform on the accuracy of predicting a woman’s non-attendance by only knowing her neighborhood of residence. However, we need to be aware that in Model 1b the neighborhood differences are possibly confounded by individual factors related to the selective migration of individuals to different neighborhoods. Because of this demographic and socioeconomic geographical segregation the neighborhood prediction would not only express contextual influences but would also be a proxy for individual characteristics.

To calculate the AU-ROC we obtained the predicted logit according to the model equation, which in Models 1b, 2 and 3 also included the neighborhood intercepts from the multilevel regression analysis. The AU-ROC measures the ability of the model to correctly classify individuals with or without the outcome (e.g., non-attendance). The AU-ROC assumes a value between 1 and 0.5 where 1 is perfect discrimination and 0.5 would indicate that the model is as informative as flipping a coin [27].

In multilevel Models 1b, 2 and 3 we estimated observational *general contextual effects* by means of the intraclass correlation coefficient (ICC). The ICC expresses the share of the total individual variance in the propensity of non-attendance that is at the neighborhood level. We expressed the ICC as a percentage and computed it according to the threshold latent variable method [20] were the individual variance is π^2^/3 and V_n_ the neighborhood variance:

$$ICC=\left[\frac{V_{n}}{V_{n}+\frac{\pi^{2}}{3}}\right]*100$$

We also computed *the median odds ratio (MOR)* [20, 34, 35] as follows:

$$MOR\approx \exp\;(0.95*\sqrt{V_{n})}$$

Even if both the ICC and the MOR are based on the same neighborhood variance, the MOR is conceptually a measure of heterogeneity, rather than of clustering as the ICC is defined above. The MOR is an alternative way of expressing neighborhood variance from a probabilistic perspective. The MOR translates the neighborhood variance to the widely used odds ratio (OR) scale, which makes the MOR comparable with the OR of individual or neighborhood variables. The MOR is defined as the median value of the distribution of ORs obtained when randomly picking two women from different neighborhoods, and comparing the one from the higher risk neighborhood to the one from the lower risk neighborhood. In simple terms, the MOR could be interpreted as the increased (median) odds of non-attendance if a woman moves to another neighborhood with higher risk. In the absence of variation, the MOR is equal to 1. The higher the MOR the more relevant is the neighborhood for understanding women’s non-attendance.

To assess to which extent a possible neighborhood variance (Vn) was explained by the individual RS (Model 2) and by the neighborhood sociodemographic index (Model 3) we quantified the proportional change of the neighborhood variance (PCV) of the initial model explained by the more advanced model. For instance, the PCV for Model 2 using Model 1b as reference would be

$$PCV=(V_{n\;model\;1b}-V_{n\;model\;2})/V_{n\;model\;1b}$$

And for Model 3 compared with Model 2

$$PCV=(V_{n\;model\;2}-V_{n\;model\;3})/V_{n\;model\;2}$$

We obtained adjusted rates of neighborhood non-participation from the shrunken residuals in Model 2. To estimate specific individual and contextual effects we calculated ORs and their 95 percent confidence intervals (95% CI). As a measure of goodness of fit of our models we use the Bayesian deviance information criterion (BDIC) [36], where the idea is that models with smaller BDIC should be preferred to models with larger BDIC. For the estimation of models we first used the restricted generalized least square (RIGLS) method to obtain start values for the final Markov chain Monte Carlo (MCMC) estimations [37]. Analyses were performed using SPSS version 20 (SPSS Inc., Chicago, IL, USA), STATA (StataCorp. 2013. Stata Statistical Software: Release 13. College Station, TX: StataCorp LP), and MLwiN version 2.22 (The Centre for Multilevel Modelling, University of Bristol, Bristol, UK).

### Ethics statement

This study was approved by the local ethics committee at Lund University (Dnr 2009/702). Active informed consent was waived as a requirement for the construction of the database. The public was informed about the implementation of the study through an advertisement in a local newspaper.

## Results

During the study period a total of 69,936 mammography screening invitations were sent out to women aged 48–75 years in Malmö. Non-attendance for the whole period was 16.3% and varied slightly across years (Table 1). The cohort selected for this study consisted of 29,915 women invited to screening during the period 2005 to 2009. Among these, 18.3% did not attend their most recent invitation. They had received on average six invitations each (range: 1–15), and 14.2% were first-time screenees. Overall the crude prevalence of non-attendance in the 212 neighborhoods averaged 17.1% and ranged from 4.3% to 47.0%. The crude prevalence obtained from the multilevel logistic regression analysis of Model 1b (the empty model) was 4.7% (range: 3.1–7.1) and the adjusted prevalence obtained from Model 2 was 3.6%, ranging from 3.2% to 7.1%.

**Table 1:** Yearly number of invitations and percentage of non-attendance among women 48–75 years of age invited to Malmö mammography screening program between 2005 and 2009.

Year	Total register		After exclusions*	
	InvitationsN	Non-attendance%	Invitations N	Non-attendance%
2005	13,679	16.5	12,954	15.9
2006	18,347	16.3	17,246	15.3
2007	15,454	17.3	14,319	15.7
2008	11,337	14.1	10,419	12.7
2009	11,119	17.1	9,756	15.7
2005–2009	69,936	16.3	64,694	15.1

* According to Fig 1, except the last step (SAMS-areas with less than 50 residents in our cohort).

Descriptive sample characteristics by quintiles of the neighborhood sociodemographic index are presented in Table 2. The median number of women per neighborhood varied between 94 and 165 across quintiles. With increasing quintile there was a consistent increase in the proportion of women who were non-attenders (Q1 = 10.9% to Q5 = 30.4%), who had low education (Q1 = 17.6% to Q5 = 36.2%), who were not employed (Q1 = 25.5% to Q5 = 51.9%), and who had spent ≤5 years in Sweden (Q1 = 0.2% to Q5 = 2.1%). The proportion of women with the highest income decreased steadily across quintiles of neighborhoods (Q1 = 31.3% to Q5 = to 11.7%), as did the proportion of women who were born in Sweden (Q1 = 88.4% to Q5 = 46.4%). Median age and number of invitations to mammography screening was lower in Q5 compared to lower quintiles, whereas the proportion of women having migrated across the Swedish boarder more than once was highest in Q4 and Q5.

**Table 2:** Characteristics of the population of women aged 48–75 years and invited to mammography screening in Malmö, Sweden, between 2005 and 2009, by quintiles of percentage of the neighborhood sociodemographic index in the population in Malmö. Values are percentages, unless otherwise indicated.

		Percentage of neighborhood sociodemographic index (quintiles)				
	Total	1^st^ quintile	2^nd^ quintile	3^rd^ quintile	4^th^ quintile	5^th^ quintile
Number of neighborhoods	212	43	42	43	42	42
Number of women	29915	4452	5171	7021	6521	6750
Median number of women per neighborhood (Min-Max)	140 (50–630)	92 (50–232)	113 (56–571)	152 (50–431)	160 (51–429)	144 (51–630)
Non-attendance	18.32	10.87	12.59	14.86	19.12	30.44
Median number of invitations (Min-Max)	5 (1–15)	5 (1–15)	6 (1–14)	6 (1–14)	5 (1–14)	4 (1–14)
Number of invitations						
< = 4	46.68	43.96	42.37	40.62	46.66	58.10
>4	53.32	56.04	57.63	59.38	53.34	41.90
Year						
2005	29.95	3.77	3.87	4.37	4.88	5.23
2006	4.50	6.42	7.17	7.89	7.81	8.07
2007	7.57	25.38	25.53	25.08	26.62	28.93
2008	26.41	32.93	32.84	32.73	30.88	29.14
2009	31.57	31.49	30.59	29.92	29.81	28.62
Season						
Fall	39.40	40.23	41.44	38.38	38.35	39.36
Winter	26.63	25.97	25.18	26.55	27.68	27.26
Spring	24.14	24.53	23.28	25.08	23.55	24.10
Summer	1.69	9.28	10.09	9.98	10.41	9.27
Age Median (Min-Max)	58 (48–75)	59 (48–75)	59 (48–75)	60 (48–75)	58 (48–75)	56 (48–75)
Age group						
48–50	14.09	14.24	12.18	11.81	14.08	17.85
51–55	24.34	22.66	22.82	20.87	23.86	30.67
56–60	18.85	18.35	19.07	19.78	19.43	17.47
61–65	16.03	17.00	17.02	17.39	16.38	12.86
66–70	12.44	13.50	13.50	13.94	11.69	10.07
71–75	14.26	14.24	15.41	16.21	14.57	11.08
Marital status						
Married	52.17	74.98	61.15	48.70	42.86	42.87
Widow	7.14	5.26	6.75	7.46	8.04	7.48
Divorced	26.83	12.42	20.32	28.19	32.31	34.62
Never married	13.85	7.35	11.78	15.65	16.79	15.02
Number of children						
0	65.49	58.13	65.21	71.60	68.67	61.11
1	19.33	19.41	19.28	18.16	19.31	20.58
>1	15.18	22.46	15.51	10.24	12.02	18.31
Education (years)						
>12	29.63	43.73	31.91	30.81	29.75	17.26
10–12	26.64	38.19	44.25	43.09	42.28	41.56
< = 9	42.04	17.57	23.42	25.41	26.82	36.18
Missing	1.69	0.52	0.43	0.70	1.15	5.01
Income (100 SEK)						
High (≥1697)	24.74	31.33	28.39	29.57	25.64	11.69
Middle-Upper (1233–1696)	24.95	24.98	24.66	26.93	26.62	22.07
Middle-Low (924–1232)	25.23	22.69	25.45	22.69	24.40	30.19
Low (<923)	25.08	21.00	21.50	20.81	23.34	36.04
Not employed	36.17	25.45	30.26	32.55	35.76	51.94
Country of birth						
Sweden	73.16	88.41	84.70	82.19	71.54	46.43
Nordic	3.60	2.81	3.17	3.59	4.32	3.76
Europe	16.39	6.65	9.55	11.04	17.64	32.40
Other	6.85	2.13	2.57	3.18	6.50	17.41
Country of citizenship						
Sweden	93.85	97.17	97.10	96.61	94.17	85.97
Nordic	1.80	1.19	1.35	1.77	2.21	2.19
Europe	2.79	1.10	1.28	1.17	2.68	6.84
Other	1.56	0.54	0.27	0.46	0.94	4.99
Missing	0.04	0	0	0.01	0.02	0.15
Time in Sweden (years)						
>5	98.58	99.06	98.98	98.93	98.65	97.53
< = 5	0.81	0.22	0.21	0.48	0.75	2.06
Missing	0.61	0.72	0.81	0.58	0.60	0.41
Migration across Swedish boarder more than once	4.67	4.04	3.79	4.71	4.95	5.45

Due to the large study sample the confidence intervals were narrow and not included in the table.

### Analysis of associations

Table 3 presents a multilevel analysis showing the specific associations between individual and neighborhood characteristics and mammography non-attendance in the study period as well as the analysis of variance and the values of the AU-ROC for the different models. As expected, there was a strong association between individual RS and non-attendance that was rather similar in all models (i.e., 1a, 2 and 3). We also observed that, independently of the individual characteristics, non-attendance increased with increasing neighborhood sociodemographic index.

**Table 3:** Multilevel analysis showing the specific associations between individual and neighborhood characteristics and mammography non-attendance during 2005–2009 among women aged 48–75 years residing in Malmö at the time of invitation (n = 29,901). Values are given as odds ratios (ORs) and 95% confidence intervals (CIs).

	Model 1a (single level)	Model 1b (empty model)	Model 2	Model 3
			OR (95% CI)	OR (95% CI)
Individual risk score^a^				
Decile group 1 (lowest risk)	Reference		Reference	Reference
Decile group 5	2.96 (2.41–3.64)		2.90 (2.31–3.49)	2.87 (2.25–3.63)
Decile group 10 (highest risk)	28.08 (23.26–33.89)		24.42 (19.62–29.26)	23.17 (18.22–29.24)
Neighborhood sociodemographic index^b^				
Decile group 1 (lowest percentage)				Reference
Decile group 5				1.26 (1.07–1.51)
Decile group 10 (highest percentage)				1.92 (1.63–2.34)
Variance		0.303 (0.238–0.380)	0.060 (0.039–0.088)	0.028 (0.013–0.046)
PCV			80%	53%
ICC (%)		8.4 (6.7–10.3)	1.8 (1.2–2.6)	0.8 (0.4–1.4)
MOR	NA	1.69 (1.59–1.80)	1.26 (1.21–1.33)	1.17 (1.12–1.23)
AU-ROC	0.747 (0.740–0.755)	0.660 (0.652–0.668)	0.760 (0.753–0.767)	0.760 (0.753–0.767)
AU-ROC change	Reference	-0.087	0.013	0.013
BDIC	24607.35	27380.77	24352.99	24468.79
BDIC change	Reference	2773.42	-254.36	-138.56

a According to a logistic regression modeling non-attendance and including as predictor variables women’s individual number of screening invitations, year and season of screening invitation, age, marital status, number of children, education, income, employment, country of birth, country of citizenship, time lived in Sweden, and history of migration abroad (for categories see Table 2 and/or Appendix?).

b Based on proportions of the population in the neighborhoods that had low education, low income, were unemployed, were not born in Sweden and had not resided in the same neighborhood during 2001–2005.

In “a” and “b” we show only the ORs for the 5^th^ and 10^th^ decile groups using the 1^st^ decile group as reference.

OR = odds ratio; CI = confidence interval; PCV = proportional change of the neighborhood variance; ICC = intraclass correlation at the neighborhood-level; MOR = Median Odds Ratio; AU-ROC = area under the ROC-curve (receiver operating characteristics); BDIC = Bayesian deviance information criterion.

### Analysis of variance

Developing the single level analysis (Model 1a, Table 3) by adding a random intercept for the neighborhood level (Model 2) considerably improved the goodness of fit since the BDIC decreased 254 units. However, Model 2 indicates that over and above the individual RS only 1.8% (i.e., ICC = 1.8%) of the total individual differences in the propensity of non-attendance were at the neighborhood level. The MOR provided similar information telling that, in the median case, if a woman moves to a neighborhood with a higher risk of non-attendance the OR will just be 1.26. This minor general neighborhood effect was in part due to the sociodemographic characteristics of the neighborhoods since adjusting for the neighborhood sociodemographic index in Model 3 decreases the variance of Model 2 by 53%. Thereby, both the ICC and the MOR became very small (i.e., 0.8% and 1.17 respectively). However, the goodness of fit of Model 3 was worse than that of Model 2 since the BDIC only decreases by 138 units compared to Model 1a.

The multilevel analysis in Model 1b indicates that 8.4% (i.e., ICC = 8.4%) of the total individual differences in the propensity of non-attendance were at the neighborhood level. However, taking into account the individual characteristic of the women in Model 2 considerably reduced the neighborhood variance (i.e., PCV = 80%) indicating that a substantial amount of the variance between neighborhoods was due to differences in their individual composition. The goodness of fit of Model 1b with only the random intercept was 2773 units higher–and thereby much worse–than Model 1a with only individual variables.

**Fig 2:**
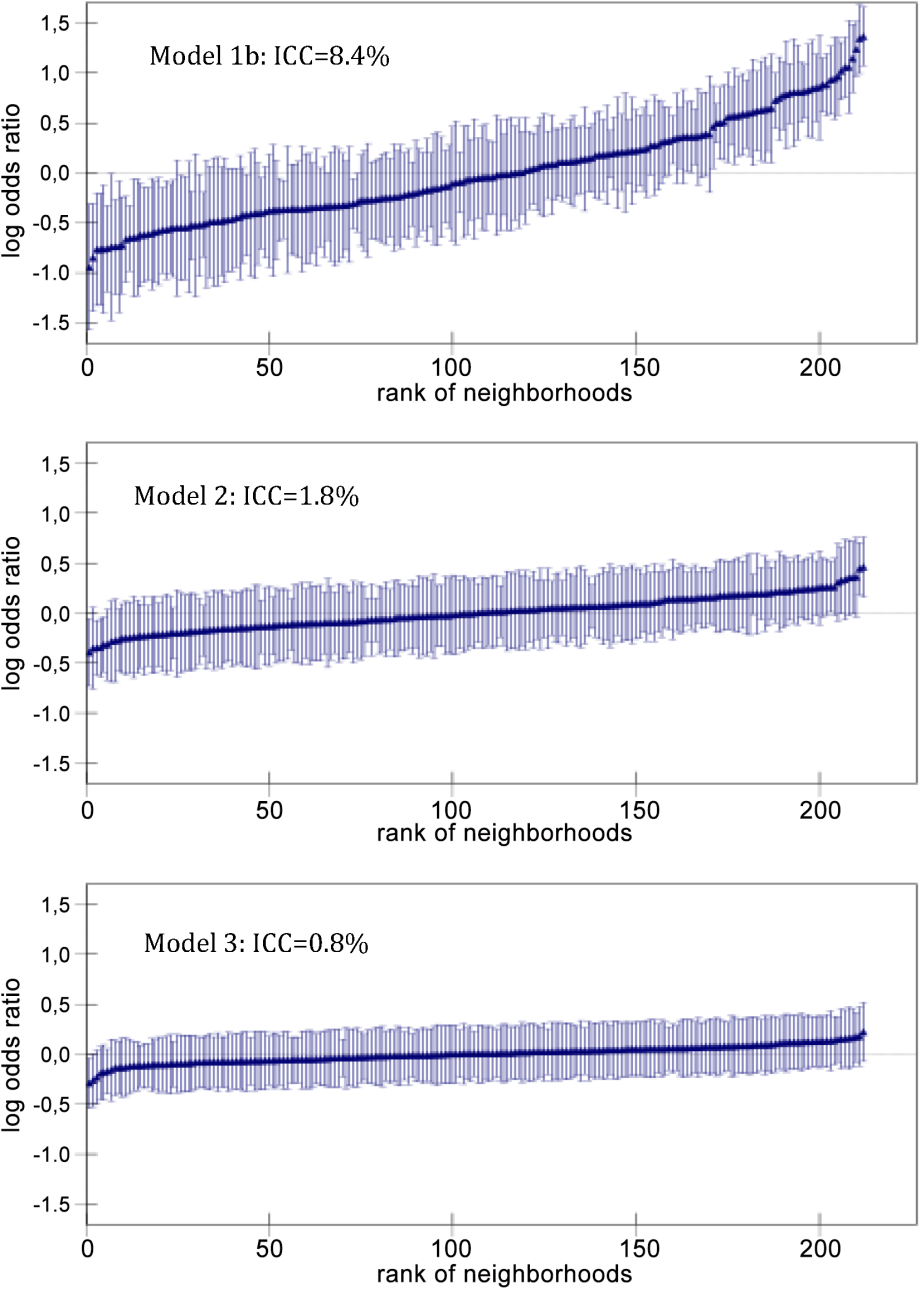
Residuals for Models 1b, 2 and 3.

The neighborhood residuals for Models 1b, 2 and 3 plotted in Fig 2 also illustrate that the initial neighborhood differences observed in Model 1b decreased considerably when taking into account the individual RS, and they were further explained by the neighborhood sociodemographic index. Remark that the neighborhood differences plotted in Fig 2 need be interpreted in conjunction with the ICC-values.

### AU-ROC analysis

As an innovative approach, our study quantified general neighborhood effects by measuring the AU-ROC. We observed that the discriminatory accuracy of considering only individual level variables (Model 1a) for classifying women according their non-attendance was 0.747, and this value showed only a minor increase (+0.013) after the inclusion of the neighborhood level as a random effect (i.e., second level) in Model 2 (AU-ROC = 0.760). That is, over and above individual level characteristics, knowledge of the neighborhoods where the women reside only provides a minor improvement for classifying women according to their non-attendance. The minor change in the AU-ROC observed in Model 2 provides the ceiling value of the AU-ROC that can be obtained by adding neighborhood level information. Therefore, the AU-ROC of Model 3 was also 0.760. Model 3 only informs us that about half (PCV = 53%) of the (small) neighborhood variance was due to the sociodemographic characteristics of the neighborhoods (i.e. the neighborhood sociodemographic index) but cannot improve the AU-ROC of Model 2 since the inclusion of the neighborhood as a random effect already covers all neighborhood information in a general, unspecified way.

**Fig 3:**
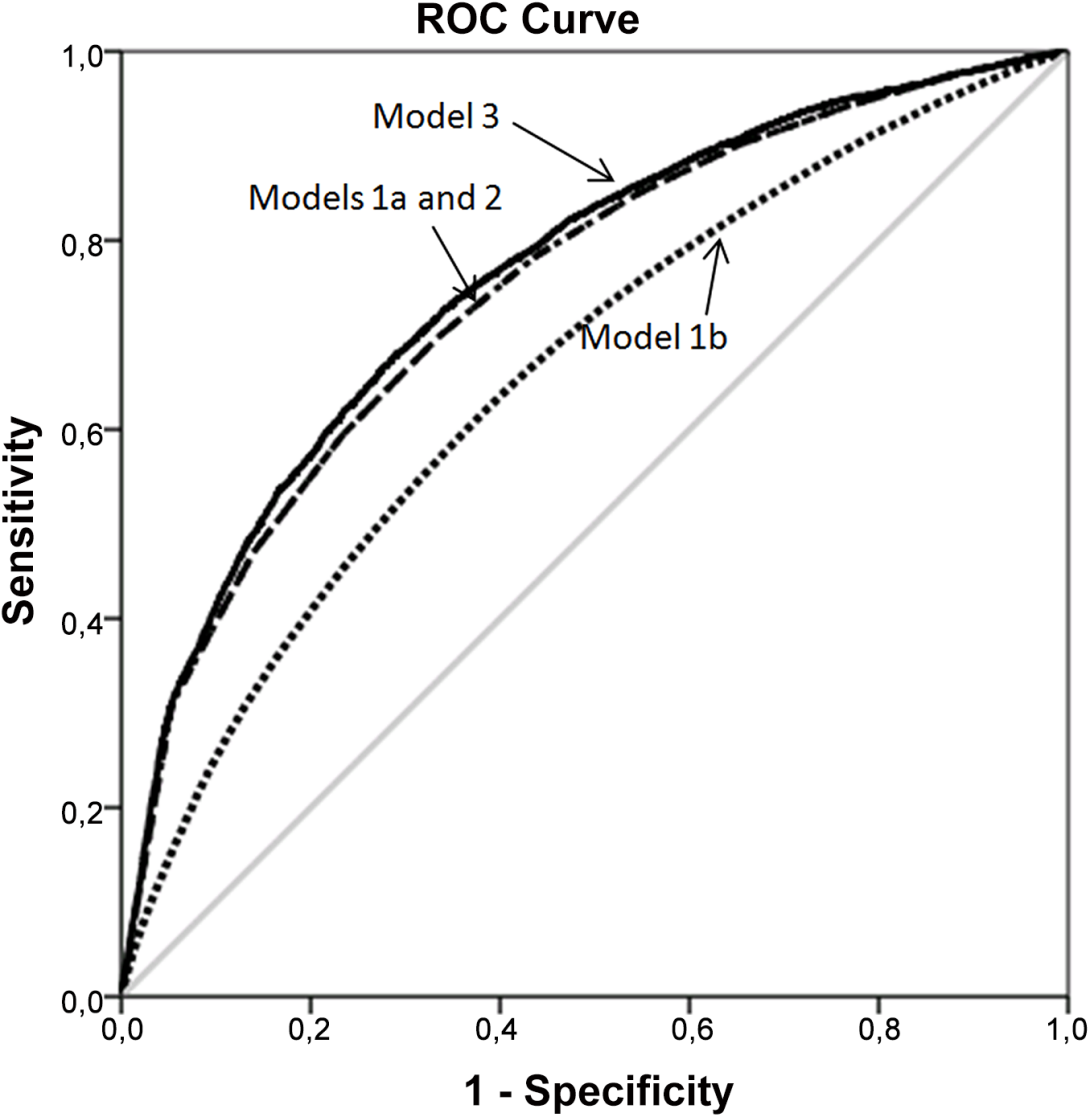
ROC-curves for Models 1a, 1b, 2 and 3.

Model 1b just has the pragmatic aim of quantifying the AU-ROC when having only neighborhood level information. The AU-ROC for this model was rather low (i.e., 0.660). Fig 3 shows the actual ROC-curves for the models discussed above.

### Characteristics of the excluded women

Table 4 shows the sociodemographic characteristics of the women who were excluded when we excluded small neighborhoods compared with the women who were included in the analyses. We found that the excluded women were younger, had fewer screening invitations, were more likely to be married, employed and born in Sweden, and more likely to have children living at home, to have more than 12 years of education and to have a high income. However, non-attendance among the selected and excluded women was similar (i.e., 18.3% versus 17.9%).

**Table 4:** Comparison between women who were included in the analytic cohort and women who were excluded (SAMS<50). Percentages are presented unless otherwise indicated.

	Included	Excluded
	*n = 29*,*915*	*n = 2468*
Women’s characteristics		
Non-attendance	18.32	17.91
Mean number of invitations (SD)	5.68 (3.67)	5.39 (3.62)
Age Mean (SD)	59.59 (8.05)	58.79 (7.80)
Married	52.17	61.91
Children living at home	34.51	40.36
>12 years of education	29.63	38.66
High income	24.74	27.51
Employed	63.83	71.84
Born in Sweden	73.16	78.44
Swedish citizenship	93.85	94.53
Lived >5 years in Sweden	99.19	99.31
Migration across Swedish border more than once	4.67	4.74

## Discussion

In this population-based register study including women 48–75 years of age invited to mammography screening in Malmö between 2005 and 2009 we found that non-attendance was 16.3% throughout the period. This is considerably lower than the non-attendance of 35% during the initial years (1990–93) of the Malmö mammography program [38], and somewhat lower than the average of 19% reported for Sweden in the past [39]. Comparing our non-attendance rate to those reported in other countries, Italy [40] and Spain [41] had lower rates, France had a higher rate [42] and the Netherland had a similar rate [43].

The prevalence rates in the multilevel logistic regression analysis (MLRA) are obtained by adding the neighborhood shrunken residual values to the overall mean in the city (i.e., the intercept in the MLRA). The neighborhood prevalences from the multilevel regression were rather different from the crude prevalence, which might puzzle some readers. However, the crude neighborhood prevalence is confounded by differences in the individual composition of the neighborhoods and also smaller neighborhoods may give extreme but uncertain values. The MLRA provides estimations that are adjusted for the individual variables and also shrunken towards the population-average by a shrinkage factor proportional to the amount of information available on each neighborhood (essentially the neighborhood size). This shrinkage is desirable as it prevents over-interpretation of otherwise extreme predictions typically associated with small neighborhoods.

### Does the neighborhood context influence women’s non-attendance at mammography screening?

In relation to the first objective of our study, among the 29,901 women and 212 neighborhoods included in the analysis we found that the neighborhood of residence had little influence on mammography non-attendance. Although we initially found a significant variation between neighborhoods, this observed general contextual effect was fairly small (i.e. 8.4%) and was decreased to 1.8% after adjusting for specific individual characteristics. This low intraclass correlation suggests that a much larger variation in the propensity for mammography non-attendance can be found within than between neighborhoods. In other words, the neighborhoods resemble random samples of the population rather than meaningful contexts conditioning women’s non-attendance. Nevertheless, over and above the individual risk score, we found a conclusive positive association between the neighborhood sociodemographic index and individual non-attendance. A similar association has previously been described in Malmö [4] and elsewhere [8, 9, 12, 14–16, 18]. However, only a limited number of previous multilevel studies examining associations between area level factors and individual level mammography attendance have simultaneously evaluated general contextual effects. After considering individual level factors a French study examining areas within the Calvados region found a similar low general contextual effect size to the one found in our study [8]. However, much larger variance was found between countries in Europe [11] and between regions across Japan [12]. A U.S. study conducted in Connecticut found a negligible and not statistically significant variance of the neighborhood random effects [14].

Our study supports findings from a previous investigation conducted by us in the city of Malmö in 1990–93 [4]. However, compared to that study the ICC of the present study is almost twice as high (8.4% vs. 4.3%). This discrepancy may represent an actual increase in the role of neighborhoods between the two time periods studied, but is more likely a consequence of differences in study design, such as definition of mammography non-attendance and the different administrative boundaries used to define neighborhoods in the two studies. Importantly, the general contextual effect of neighborhood of residence was reduced to less than 2% in both studies after adjusting for individual-level characteristics.

Our results were also well illustrated by the neighborhood MOR that was drastically reduced when adjusting for individual level characteristics. Also the AU-ROC-analysis provides relevant information that indicates that the neighborhood level had only contributed to a minor degree to the prediction of non-attendance over and above the individual risk score. This information is important to consider when interpreting the association between the neighborhood sociodemographic index and individual non-attendance.

Besides non-attendance at mammography screening, several earlier neighborhood multilevel analyses in the city of Malmö have found minor general contextual effects on different health related outcomes such as blood pressure [44], social participation [45], use of hormone replacement therapy, blood pressure lowering drugs [46], psychotropic medication [47], self-reported health [48], health-related quality of life [49], all-cause mortality and cause specific morbidity [50], and utilization of psychiatric care [51]. In these studies the neighborhood context only accounted for at most 6.3% of the total individual variance in the health outcome.

### Is knowledge of a woman’s neighborhood of residence sufficient to predict non-attendance?

One of the multilevel models (i.e. Model 1b) only contained information on neighborhoods as a random intercept since our idea was that we could use this information to identify non-attenders. From this pragmatic perspective it does not matter if the neighborhood variance is occasioned by differences in the individual composition of the neigborhoods (i.e.,the existence of confounding). However, the size of the AU-ROC for Model 2 indicates that neighborhood by itself constitutes an insufficient tool to predict mammography non-attendance.

Our results are relevant when planning public health interventions to promote mammography screening attendance. For example, policy makers aiming to improve attendance in the city of Malmö would need to realize that focusing on specific neighborhoods would not be effective because of the low discriminatory accuracy of this information. In other words, information on neighborhood of residence does not provide accurate information for identifying target groups. If policy makers do choose to focus on neighborhoods with a higher average risk of non-attendance they need to be aware that many non-attenders would be living in the “low-risk” neighborhoods and that many attenders would be living in the “high-risk” neighborhoods. That is, focusing on only the high-risk neighborhoods would unnecessarily expose many women to an intervention they do not need and would leave many women unexposed because they live in low-risk neighborhoods. Perhaps a better approach would be to launch an intervention for the whole population in Malmö.

### Strengths and limitations

Sweden has a long tradition of administrating demographic, socioeconomic and medical data in large population based registers that are regularly subject to quality controls. Both independent and dependent variables are based on register data and not on self-reported data, which decreases the risk of misclassification of both exposures and outcome. Sweden has a well-established public health care system with very high coverage. However, at the time of the study period there was one private screening clinic in Malmö and some of the women classified as non-attenders may have had mammography at such private clinics, thus disqualifying them as true non-attenders. About 10% of non-attenders and 4.5% of the attenders had undergone mammography at one of the private clinics during the period 1990–1993 [38]. Previous studies in both Sweden and other countries have found that non-attenders who attended private clinics were more likely to, for example, be of higher socioeconomic status than true non-attenders [38, 52, 53]. The fact that they were categorized as non-attenders may thus have led to an underestimation of true differences between attenders and non-attenders. Since we lacked more updated information about the number of women undergoing mammography at private clinics we were not able to investigate this issue further.

Furthermore, not all women in the area were included in this study, due to the fact that some were on a so-called ‘Do Not Invite list’ (DNI-list). One of the two major categories of women being added to this list is breast cancer patients. A breast cancer diagnosis automatically qualifies patients for clinical rather than screening mammography. In our study women who had ever had a breast cancer diagnosis were excluded from the sample anyways, so that should not have affected our results. However, the other category consists of women who have, themselves, asked not to be invited to the screening program. The vast majority of women in this category were classified as non-attenders based on their most recent invitation (data not shown). We estimate that approximately 8% of the women who were eligible for screening were on the DNI-list as of January 1, 2008. With non-attenders accumulating on the DNI-list over time the screening program is left with inviting a selection of women who are more likely to attend. This in turn means that we may have under-estimated the non-attendance rate across the study period. The increase in attendance compared to the previous study in the region [38], could however also be a result of the screening program gaining acceptance in the population, perhaps as a result of increased awareness of the risk of getting breast cancer.

We used a composite index of neighborhood sociodemographic characteristics to identify specific contextual effects. However, we did not use any of the established deprivation indices [54]. This situation could decrease the comparability of our results with other studies. However, it is well established that those indices correlated with each other to a considerable degree, so the choice of one index versus another does not have a major relevance [55].

Our neighborhood variable, SAMS, refers to area of residence at the time of the most recent invitation. These areas are arbitrary administrative geographical divisions that may not capture the place or context where behaviors of all individuals are influenced the most and may not reflect a meaningful social community as defined by residents. Other geographic areas or contexts where work, studies and other activities are taking place may therefore be more influential in shaping behaviors. However, information on SAMS is easily accessible which represents a major practical advantage. Another limitation of this study was the lack of longitudinal information on neighborhoods where women may have lived before and that may have shaped their health behaviors. However, we did consider some migratory factors in the analysis, i.e. individual history of moving in and out of Sweden and extent of the population moving in an out of the respective residential neighborhoods. According to the LOMAS database about 77% of the population in the 212 neighborhoods had stayed in the same neighborhood throughout the years 2000–2005. This indicates that there is a degree of internal movement, which theoretically may cause dilution bias and an underestimation of the neighborhood variance.

Our results are applicable mainly to urban areas, since the majority of neighborhoods in Malmö are more densely populated, especially considering that areas with less than 50 eligible women were excluded from the study population. Furthermore, our results may not be applicable to women younger than 48 years of age, since these have only been invited for screening more recently in this geographical area and were not included in our analyses.

We did not consider all possible factors that have been found to affect mammography attendance in Sweden, such as attitudes, beliefs and knowledge [56], other health-related behaviors and previous experience of cancer and breast disease [57, 58], and psychosocial factors [59]. However, the program-related and sociodemographic characteristics of women and neighborhoods that were considered in the analysis almost entirely explained the variance in mammography non-attendance between neighborhoods seen in the empty model, and would thus have sufficiently captured or represented relevant characteristics of individuals and neighborhoods for the purpose of evaluating the independent influence of neighborhoods on mammography non-attendance.

Further research should examine whether other geographical, cultural and social contexts have more influence over mammography attendance and may be more relevant for public health interventions aimed at increasing attendance.

## Conclusions

Our results suggest that the neighborhoods in Malmö, Sweden (as measured by SAMS-areas) do not seem to condition women’s participation in population based mammography screening. Therefore, information on a woman’s neighborhood of residence constitutes an inefficient tool for discriminating mammography non-attendance. Thus, targeting specific neighborhoods to increase mammography screening attendance would not be useful. Interventions should be directed to the whole city and be focused on women with high risk scores and thereby a higher risk of non-attendance. For this purpose a formal risk score equation that could be generalized to other contexts and time periods could be developed [31]. However, in the absence of easy access to information on women’s characteristics, neighborhoods with particularly low attendance could be targeted for pragmatic reasons. However, such intervention would leave many women with high risk for non-attendance unattended and would also unnecessarily address many women that would attend mammography in any case.

## Supporting Information

S1 File Merlo J, Wagner Ph, Ghith N, Leckie G. A novel stepwise multilevel logistic regression approach to analysis of individual heterogeneity using measures of discriminatory accuracy: the case of neighbourhoods and health. Manuscript submitted PLOS-ONE, 2015.
(PDF)

S1 Table Coefficients used for women’s individual risk score. Multivariable logistic regression modeling screening related variables and women’s individual sociodemographic characteristics and the odds of mammography non-attendance (n = 29,901).
(DOCX)
