# Supplementary material for: Does the Neighborhood Area of Residence Influence Non-Attendance in an Urban Mammography Screening Program? A Multilevel Study in a Swedish City
Source: PLoS One. 2015 Oct 13;10(10):e0140244. doi: 10.1371/journal.pone.0140244 (PMC4604149; doi:10.1371/journal.pone.0140244)
Supplement: S1 File — A novel stepwise multilevel logistic regression approach to analysis of individual heterogeneity using measures of discriminatory accuracy: the case of neighbourhoods and health. Manuscript submitted PLOS-ONE, 2015. (PDF) [file pone.0140244.s001.pdf]

PLOS ONE

A novel stepwise multilevel logistic regression analysis of discriminatory accuracy: the case of neighbourhoods and health

--Manuscript Draft--

**Manuscript Number:**	PONE-D-15-36083
**Article Type:**	Research Article
**Full Title:**	A novel stepwise multilevel logistic regression analysis of discriminatory accuracy: the case of neighbourhoods and health
**Short Title:**	A novel multilevel analysis of neighbourhoods and health
**Corresponding Author:**	Juan Merlo, M.D., Ph.D. Professor<br>Lund University<br>Malmo, SWEDEN
**Keywords:**	Multilevel regression analysis; variance analysis; discriminatory accuracy; neighbourhood; psychotropic medication; health care utilization; geographical differences, social epidemiology; Sweden
**Abstract:**	Background and aim: Many multilevel logistic regression analyses of "neighbourhood and health" focus on interpreting measures of associations (e.g., odds ratio, OR). In contrast, multilevel analysis of variance is rarely considered. We propose a novel stepwise analytical approach that distinguishes between "specific" (measures of association) and "general" (measures of variance) contextual effects and discuss appropriate epidemiological measures for this purpose.<br><br>Methods: We analyse 43,291 individuals residing in 218 neighbourhoods in the city of Malmö, Sweden in 2006. We study two individual outcomes (psychotropic drug use and choice of private vs. public general practitioner, GP) for which the relative importance of neighbourhood as a source of individual variation differs substantially. In Step 1 of the analysis, we evaluate the OR and the area under the receiver operating characteristic (AU-ROC) curve for individual-level covariates. In Step 2, we assess general contextual effects using the AU-ROC. Finally, in Step 3 the OR for a specific neighbourhood characteristic (e.g., neighbourhood income) is interpreted jointly with the proportional change in variance (i.e., PCV) and the proportion of ORs in the opposite direction (POOR).<br><br>Results: For both outcomes, information on individual characteristics (Step 1) provide a low discriminatory accuracy (AU-ROC=0.616 for psychotropic drugs; =0.600 for choosing a private physician). Accounting for neighbourhood of residence (Step 2) only improved the AU-ROC for choosing a private physician (+0.295 units). High neighbourhood income (Step 3) was strongly associated to choosing a private physician (OR= 3.50) but the PCV was only 11% and the POOR 33%.<br><br>Conclusion: We develop and exemplify a novel stepwise multilevel analytical approach. We observed that the neighbourhood context in Malmö had a negligible influence on individual use of psychotropic drugs, but appears to strongly condition individual choice of a private GP. However, the reasons for this phenomenon are only partially explained by the socioeconomic circumstances of the neighbourhoods.
**Order of Authors:**	Juan Merlo, M.D., Ph.D. Professor
	Philippe Wagner
	Nermin Ghith
	George Leckie
**Opposed Reviewers:**	
**Additional Information:**	
**Question**	**Response**
**Financial Disclosure**	Swedish Research Council (JM, 2013-2484)

Powered by Editorial Manager® and ProduXion Manager® from Aries Systems Corporation

Please describe all sources of funding that have supported your work. A complete funding statement should do the following:<br><br>Include **grant numbers and the URLs** of any funder's website. Use the full name, not acronyms, of funding institutions, and use initials to identify authors who received the funding.<br>**Describe the role** of any sponsors or funders in the study design, data collection and analysis, decision to publish, or preparation of the manuscript. If they had no role in any of the above, include this sentence at the end of your statement: "*The funders had no role in study design, data collection and analysis, decision to publish, or preparation of the manuscript.*"<br><br>If the study was **unfunded**, provide a statement that clearly indicates this, for example: "*The author(s) received no specific funding for this work.*"<br><br>* typeset	http://www.vr.se/inenglish.4.12fff4451215cbd83e4800015152.html<br><br>The funders had no role in study design, data collection and analysis, decision to publish, or preparation of the manuscript."
**Competing Interests**<br><br>You are responsible for recognizing and disclosing on behalf of all authors any competing interest that could be perceived to bias their work, acknowledging all financial support and any other relevant financial or non-financial competing interests.<br><br>Do any authors of this manuscript have competing interests (as described in the PLOS Policy on Declaration and Evaluation of Competing Interests)?<br><br>**If yes**, please provide details about any and all competing interests in the box below. Your response should begin with this statement: *I have read the journal's policy and the authors of this manuscript have the following competing interests:*<br><br>**If no** authors have any competing interests to declare, please enter this	The authors have declared that no competing interests exist

statement in the box: "*The authors have declared that no competing interests exist.*"<br><br>* typeset	
**Ethics Statement**<br><br>You must provide an ethics statement if your study involved human participants, specimens or tissue samples, or vertebrate animals, embryos or tissues. All information entered here should **also be included in the Methods section** of your manuscript. Please write "N/A" if your study does not require an ethics statement.<br><br>**Human Subject Research (involved human participants and/or tissue)**<br><br>All research involving human participants must have been approved by the authors' Institutional Review Board (IRB) or an equivalent committee, and all clinical investigation must have been conducted according to the principles expressed in the Declaration of Helsinki. Informed consent, written or oral, should also have been obtained from the participants. If no consent was given, the reason must be explained (e.g. the data were analyzed anonymously) and reported. The form of consent (written/oral), or reason for lack of consent, should be indicated in the Methods section of your manuscript.<br><br>Please enter the name of the IRB or Ethics Committee that approved this study in the space below. Include the approval number and/or a statement indicating approval of this research.<br><br>**Animal Research (involved vertebrate animals, embryos or tissues)**<br><br>All animal work must have been conducted according to relevant national and international guidelines. If your study involved non-human primates, you must provide details regarding animal welfare and steps taken to ameliorate suffering; this is in accordance with the recommendations of the Weatherall report, "The use of non-human primates in research." The relevant guidelines	The National Board of Health and Welfare and Statistics Sweden constructed the database by means of record linkage of different registers using the unique Swedish personal identification number. Finally, the Swedish authorities delivered the research database to us without the personal identification numbers to ensure the anonymity of the subjects. The Regional Ethics Review Board in southern Sweden as well as the data safety committees from the National Board of Health and Welfare and from Statistics Sweden approved the construction of the LOMAS database.<br>For the purpose of our study we created a fully anonymized sample that completely prevents the identification of individuals using a combination of variables. This fully anonymized sample is provided in the Online Supplementary Materials.

Powered by Editorial Manager® and ProduXion Manager® from Aries Systems Corporation

followed and the committee that approved the study should be identified in the ethics statement.<br><br>If anesthesia, euthanasia or any kind of animal sacrifice is part of the study, please include briefly in your statement which substances and/or methods were applied.<br><br>Please enter the name of your Institutional Animal Care and Use Committee (IACUC) or other relevant ethics board, and indicate whether they approved this research or granted a formal waiver of ethical approval. Also include an approval number if one was obtained.<br><br>**Field Permit**<br><br>Please indicate the name of the institution or the relevant body that granted permission.	
**Data Availability**<br><br>PLOS journals require authors to make all data underlying the findings described in their manuscript fully available, without restriction and from the time of publication, with only rare exceptions to address legal and ethical concerns (see the PLOS Data Policy and FAQ for further details). When submitting a manuscript, authors must provide a Data Availability Statement that describes where the data underlying their manuscript can be found.<br><br>Your answers to the following constitute your statement about data availability and will be included with the article in the event of publication. **Please note that simply stating 'data available on request from the author' is not acceptable.** ***If, however, your data are only available upon request from the author(s), you must answer "No" to the first question below, and explain your exceptional situation in the text box provided.***<br><br>Do the authors confirm that all data underlying the findings described in their manuscript are fully available without restriction?	Yes - all data are fully available without restriction
Please describe where your data may be found, writing in full sentences. **Your answers should be entered into the box below and will be published in the form**	All relevant data are within the paper and its Supporting Information files

**you provide them, if your manuscript is accepted.** If you are copying our sample text below, please ensure you replace any instances of XXX with the appropriate details.<br><br>If your data are all contained within the paper and/or Supporting Information files, please state this in your answer below. For example, “All relevant data are within the paper and its Supporting Information files.”<br>If your data are held or will be held in a public repository, include URLs, accession numbers or DOIs. For example, “All XXX files are available from the XXX database (accession number(s) XXX, XXX)." If this information will only be available after acceptance, please indicate this by ticking the box below.<br>If neither of these applies but you are able to provide details of access elsewhere, with or without limitations, please do so in the box below. For example:<br><br>“Data are available from the XXX Institutional Data Access / Ethics Committee for researchers who meet the criteria for access to confidential data.”<br><br>“Data are from the XXX study whose authors may be contacted at XXX.”<br><br>* typeset	
Additional data availability information:	Tick here if your circumstances are not covered by the questions above and you need the journal’s help to make your data available.

Powered by Editorial Manager® and ProduXion Manager® from Aries Systems Corporation

Dear Editor,

An established area of research in social epidemiology and public health concerns the investigation of "neighbourhood and health" [1]. Researchers aim to identify contextual influences of the neighbourhood of one's residence on individual health outcomes. For this purpose, multilevel analysis of variance is a fundamental methodology that allows appropriate measurement and interpretation of contextual effects [2, 3]. However, many studies continue to focus on estimating and interpreting only measures of association (e.g., odds ratios) between specific contextual characteristics and individual health outcomes [4]. Unfortunately, this may lead to incorrect inferences and, thereby, incorrect conclusions. Though, multilevel analysis of variance is technically more complex than standard analyses and this may have deterred many researchers from applying it. This research article therefore proposes an innovative and accessible three-step approach to conducting multilevel analysis of variance in neighbourhood and health studies. Our approach distinguishes between "specific" (measures of association) and "general" (measures of variance) contextual effects. We provide and compare different measures of (observational) contextual effects and introduce the area under the receiver operating characteristic curve (AU-ROC) as an intuitive measure to quantify general contextual effects.

While our contribution is fundamentally methodological, we illustrate our three-step approach by performing real empirical analyses paying special attention to describe and explain the applied methodology. Furthermore, we present our ideas in a didactic and conceptual fashion, rather than a mathematical one, in order to make our arguments and methods accessible to as broad a readership as possible.

Our team has considerable experience in the analysis and interpretation of multilevel analyses of variance and we have also published several tutorials on other aspects of multilevel modelling. We believe our study fills a gap in the current literature on multilevel analysis and it will therefore be received with interest by many researchers. We hope that our work will facilitate and improve the use and interpretation of multilevel regression analyses in Public Health which, in turn, will ultimately lead to improvements in public health practice.

Sincerely

Juan Merlo, on behalf of all the authors

1. Kawachi, I. and L. Berkman, *Neighborhoods and Health*. 2003, New York: Oxford University Press.
2. Merlo, J., *Invited commentary: multilevel analysis of individual heterogeneity-a fundamental critique of the current probabilistic risk factor epidemiology.* American Journal of Epidemiology, 2014. **180**(2): p. 208-12.
3. Riva, M., L. Gauvin, and T.A. Barnett, *Toward the next generation of research into small area effects on health: a synthesis of multilevel investigations published since July 1998.* J Epidemiol Community Health, 2007. **61**(10): p. 853-61.
4. Diez Roux, A.V., *Next steps in understanding the multilevel determinants of health.* J Epidemiol Community Health 1470-2738 (Electronic), 2008. **62**(11): p. 957-9.

Manuscript
Click here to download Manuscript: r1_Final_Paper_MLAUROC_150813.docx

A novel stepwise multilevel logistic regression analysis of discriminatory accuracy: the case of neighbourhoods and health

Short title: A novel multilevel analysis of neighbourhoods and health

Juan Merlo[1], Philippe Wagner[1,2,], Nermin Ghith[1,3,] George Leckie[4]

[1]Unit for Social Epidemiology, Faculty of Medicine, Lund University, Malmö, Sweden

[2]Centre for Clinical Research Västmanland, Uppsala University, Uppsala, Sweden

[3]Research Unit of Chronic Conditions, Bispebjerg University Hospital, Copenhagen, Denmark

[4]Centre for Multilevel Modelling, University of Bristol, UK

*Corresponding author

E-mail: juan.merlo@med.lu.se

Abstract

Background and aim: Many multilevel logistic regression analyses of "neighbourhood and health" focus on interpreting measures of associations (e.g., odds ratio, OR). In contrast, multilevel analysis of variance is rarely considered. We propose a novel stepwise analytical approach that distinguishes between "specific" (measures of association) and "general" (measures of variance) contextual effects and discuss appropriate epidemiological measures for this purpose.

Methods: We analyse 43,291 individuals residing in 218 neighbourhoods in the city of Malmö, Sweden in 2006. We study two individual outcomes (psychotropic drug use and choice of private vs. public general practitioner, GP) for which the relative importance of neighbourhood as a source of individual variation differs substantially. In Step 1 of the analysis, we evaluate the OR and the area under the receiver operating characteristic (AU-ROC) curve for individual-level covariates. In Step 2, we assess general contextual effects using the AU-ROC. Finally, in Step 3 the OR for a specific neighbourhood characteristic (e.g., neighbourhood income) is interpreted jointly with the proportional change in variance (i.e., PCV) and the proportion of ORs in the opposite direction (POOR).

Results: For both outcomes, information on individual characteristics (Step 1) provide a low discriminatory accuracy (AU-ROC=0.616 for psychotropic drugs; =0.600 for choosing a private physician). Accounting for neighbourhood of residence (Step 2) only improved the AU-ROC for choosing a private physician (+0.295 units). High neighbourhood income (Step 3) was strongly associated to choosing a private physician (OR= 3.50) but the PCV was only 11% and the POOR 33%.

Conclusion: We develop and exemplify a novel stepwise multilevel analytical approach. We observed that the neighbourhood context in Malmö had a negligible influence on individual use of psychotropic drugs, but appears to strongly condition individual choice of a private GP. However, the reasons for this phenomenon are only partially explained by the socioeconomic circumstances of the neighbourhoods.

Introduction

An established area of research in social epidemiology and public health concerns the investigation of "neighbourhood and health" and multilevel logistic regression analyses are frequently conducted for this purpose. Interest within neighbourhood and health studies typically lies in estimating and interpreting measures of associations (e.g., the exponentiated regression coefficients or odds ratios, OR) between specific contextual characteristics and binary measures of individual health outcomes. In other settings, researchers routinely perform analyses of small area variation which, in their simplest form, are displayed as health league tables, "heat" or choropleth maps, or atlases of geographical variation. A common denominator in all these studies is that they analyse differences between group averages. For instance, the average risk of dying among individuals living in poor neighbourhoods might be compared to the average risk of dying among individuals living in rich neighbourhoods. Alternatively, statistics like indices of small area variation might be calculated to summarize the overall range or variation in group averages. All these studies disregard within-group individual-level variation in health outcomes except to estimate the statistical uncertainty around the estimated differences between group averages (1, 2).

In contrast, other researchers have explicitly concluded that we need to consider both differences between group averages and differences between individuals around these averages. In fact, information on individual-level variance in multilevel regression analysis provides indispensable information for understanding contextual influences on health (1-9). From this perspective, knowing the proportions of overall variation in health outcomes which are attributable to the contextual-level (e.g., the neighbourhood) is of fundamental relevance for operationalizing contextual phenomena and for identifying the relevant levels of analysis (1, 3, 7, 10-14). This concept is rather intuitive when we think about the analogy between individual and collective bodies (3). Also, using Rose's terminology (15), in order to identify

sick populations the simple quantification of differences between population averages of some health indicator is not appropriate. Rather, we need information on both population averages and the distribution of individual values around these averages. Through doing so, we are able to learn the share of the total outcome variance that is between population level averages so the larger this proportion, the more relevant the population level of analysis is (7). This idea corresponds well with the notion of variance partition coefficients (VPC) and the concept of clustering as measured by intraclass correlation coefficients (ICC) (12).

Considering these ideas, we can identify at least three different analytical approaches in social epidemiology, all of which are dedicated to the investigation of contextual influences on binary measures of individual health. The *small area variation approach* focuses on the analysis of geographic variance using aggregated geographical data often on small areas or zones at different spatial scales (16). The *multilevel analysis of associations approach* performs multilevel logistic regression analysis or similar techniques to identify average associations (e.g., ORs) between specific contextual level variables and individual health (17), adjusting for neighbourhood clustering. Finally, the *multilevel analysis of individual heterogeneity approach* combines both the multilevel analysis of associations for estimation of specific contextual effects and the multilevel analysis of variance (e.g., the degree of clustering, ICC) for the investigation of general contextual effects (i.e., non-specific contextual influences on health) (3) (2). The *small area variation approach* typically applied in Public Health represents a refinement of classical ecological studies on aggregated data. The *multilevel analysis of associations approach* follows the conventional approach in probabilistic risk factors epidemiology, while the *multilevel analysis of individual heterogeneity approach* adopts a multilevel perspective for understanding heterogeneity of individual responses around the average risk in a group (18). It is this last approach which we develop and promote in this study.

Interestingly, in spite of their independent origins and areas of application, the *multilevel analysis of individual heterogeneity approach* has many analogies with that adopted in other fields of epidemiology concerned with the identification of new candidate risk factors and biomarkers and the evaluation of diagnostic and screening test. In those research fields, it is well known that measures of average association like ORs provide limited information for gauging the performance of a diagnostic, prognostic, or screening marker (19). Accordingly, the rule is that measures of association need be interpreted together with measures of discriminatory accuracy such as net reclassification improvement (NRI), integrated discrimination improvement (IDI) (20-22) and, especially, the area under the receiver operating characteristic curve (AU-ROC) (23, 24). Analogously, the *multilevel analysis of individual heterogeneity approach* argues that estimates of specific contextual effects (i.e., average measures of association) provide insufficient information if they are not accompanied by measures of general contextual effects (i.e., degree of clustering) (1, 2, 18).

In the *multilevel analysis of individual heterogeneity approach* the ICC for hierarchical multilevel structures (25) is a fundamental measure for quantifying general contextual effects. As a concept, the ICC (i.e., the share of the total outcome variance which lies at the context level, having adjusted for any covariates) is rather intuitive for continuous responses since the individual- and contextual-level variances are both estimated and defined on the same scale. However, the ICC proves less straightforward to understand and calculate when analysing binary responses via multilevel logistic regression because only the contextual-level variance is estimated. Furthermore, this variance is defined on the log-odds scale, rather than the binary response scale (25). Nevertheless, a range of procedures for calculating the ICC for binary responses have been proposed, including the normal approximation, the simulation method, and the Taylor series linearization, (10, 25-27). However, it is the ICC based on the latent response formulation of the model which has become most widely adopted. No doubt partly

due to these complications, a range of alternatives to the ICC for binary responses have also been proposed to quantify the extent of general contextual effects. These include the pairwise odds ratio (PWOR)(14) and measures of heterogeneity such as the median odds ratio (MOR)(28, 29). In any case, it is important to realize that the ICC is itself a measure of discriminatory accuracy (30, 31). Therefore, taking advantage of the analogy between the concept of discriminatory accuracy and the notion of general contextual effects, a simple but innovative approach is to express general contextual effects by means of measures of discriminatory accuracy like the AU-ROC (32, 33). The AU-ROC measure is well established among epidemiologists, public health practitioners and physicians and its computation is straightforward using standard statistical software.

In the current study, we present a novel three-step approach for the systematic investigation of observational multilevel (e.g., individual and neighbourhood) effects on binary measures of individual health and health care utilization, distinguishing between specific and general contextual effects. To make our approach as accessible as possible, we present a conceptual and didactic treatment of the issues rather than a technical and mathematical one. We introduce and then demonstrate the utility of AU-ROC as a measure of general contextual effects and we compare it to the ICC and the MOR. We illustrate our approach by analysing two different binary outcomes: (i) use of psychotropic medication, which is related to both psychological health and access to medication; and (ii) individual choice of a private vs. a public general practitioner (GP), which is a behavioural outcome.

Population and methods

Study sample

We drew our sample of individuals from the LOMAS (Longitudinal Multilevel Analysis in Scania) database containing anonymised data on all individuals living in the county of Scania,

Sweden during the years 1968-2006. The database includes geographic, demographic and socioeconomic information on all individuals as well as data on their health care and medication use (34). The sample consists of all individuals aged 35–64 years residing in the city of Malmö on 31st December 2005 (N= 99,266), who were still alive on 31st December 2006 (N = 98,536). We further restricted this sample to those with at least one contact with primary health care during the year 2006 (N = 46,675) as well as residing in neighbourhoods with at least 50 people who fulfilled the same selection criteria (N = 43,588). Lastly, we dropped 297 (0.7%) individuals who had missing values for individual income. The final study sample consisted of 43,291 individuals within 218 neighbourhoods. This dataset (fully anonymized) is provided in the Online Supplementary Materials.

The National Board of Health and Welfare and Statistics Sweden constructed the database by means of record linkage of different registers using the unique Swedish personal identification number. Finally, the Swedish authorities delivered the research database to us without the personal identification numbers to ensure the anonymity of the subjects. The Regional Ethics Review Board in southern Sweden as well as the data safety committees from the National Board of Health and Welfare and from Statistics Sweden approved the construction of the LOMAS database.

For the purpose of our study we created a fully anonymized sample that completely prevents the identification of individuals using a combination of variables. This fully anonymized sample is provided in the Online Supplementary Materials.

Assessment of variables

Outcome variables

To illustrate our three-step approach, we carried out two empirical analyses. In the first analysis the outcome variable was defined as use (= 1) or not (= 0) of psychotropic medication during 2006. We defined psychotropic medication as Anatomical Therapeutic Chemical (ATC) Classification System (35) codes N05B (Anxiolytics), N05C (Hypnotics and sedatives) and N06A (Antidepressants). In the second analysis, the response variable was whether a person had visited a private (=1) or public (= 0) specialist physician in general practice (GP) during the year.

Individual characteristics

In order to illustrate our approach as clearly as possible, we considered only three individual-level covariates: age categorized into five age groups, 35–39, 40–45, 50–54, 55–59, and 60–65 years, using the youngest age group as the reference category in the model specifications; sex that compared men (=1) with women (=0); and income categorized as ‘low’ when having less that the median income in Malmö, or ‘high’ otherwise. In the analysis of psychotropic medication the reference category was high income while in the analysis of private GP choice the reference category was low income. These choices are cosmetic, but ensure that we estimate positive rather than negative associations between the outcome and income which are easier for readers to interpret (psychotropic medication use is higher among the poor while private GP use is higher among the rich). The median income in Malmö was derived from individualized household disposable income in 2004 for all individuals aged 35 to 85 in the city.

Neighbourhood variables

We defined *neighbourhoods* using small-area market statistics (SAMS) boundaries created by Statistics Sweden (36). The SAMS boundaries are based on municipalities' sub-division boundaries which are constructed to maximise the internal homogeneity of housing tenure. The resulting neighbourhoods have an average population of around 1000 individuals.

For simplicity, we categorized neighbourhoods as 'rich' or 'poor' according to whether the proportion of low income individuals in each neighbourhood was below the median across all neighbourhoods in the city. Paralleling the way we entered individual income into our models, in the analysis of psychotropic medication we set the reference category for neighbourhood income to be rich neighbourhoods while in the analysis of private GP we set the reference category to be poor neighbourhoods.

Multilevel analysis of heterogeneity

The data have a two-level hierarchical structure with individuals (level 1) nested within neighbourhoods (level 2). For the analysis we applied a three step-approach consisting of fitting, interpreting and contrasting the results of three consecutive multilevel logistic regression models: the individual effects model (Step 1); the general contextual effects model (Step 2); and the specific contextual effects model (Step 3).

Let y_{ij} denote the binary response of interest (e.g., use of psychotropic medication or private GP) for individual i ($i = 1, \dots, n_j$) in neighbourhood j ($j = 1, \dots, J$).

Step 1 - The individual effects model: Step 1 simply consists of fitting a conventional single-level logistic regression for y_{ij} including only the individual-level covariates; neighbourhoods are completely ignored. In terms of our two illustrative applications, the covariates are age, sex and income. The model is therefore written as

$$y_{ij} \sim \text{Binomial}(1, \pi_{ij}), \tag{1}$$

$$\text{logit}(\pi_{ij}) = \beta_0 + \beta_1\texttt{age}_{ij} + \beta_2\texttt{sex}_{ij} + \beta_3\texttt{income}_{ij},$$

where π_{ij} denotes the probability that individual i in neighbourhood j uses psychotropic medication (or private GP) given their individual characteristics $\texttt{age}_{ij}$, $\texttt{sex}_{ij}$ and $\texttt{income}_{ij}$. The regression coefficients $\beta_1, \beta_2, \beta_3$ measure the associations between the log-odds of the health outcome and each covariate all else equal and when exponentiated these are translated to ORs. For ease of illustration we have entered age into the model linearly, but we shall relax this assumption when we fit the model. Post-estimation, predicted probabilities $\hat{\pi}_{ij}$ are calculated for each individual and are used to calculate the AU-ROC for the model.

The AU-ROC (32, 33) is constructed by plotting the true positive fraction (TPF) (i.e., sensitivity) against the false positive fraction (FPF) (i.e., 1 − specificity) for different binary classification thresholds of the predicted probabilities. The AU-ROC measures the ability of the model to correctly classify individuals with or without the outcome (e.g., using or not psychotropic medication or visiting a private vs. a public GP) as a function of individuals' predicted probabilities. The AU-ROC takes a value between 1 and 0.5 where 1 is perfect discrimination and 0.5 would be as equally as informative as flipping a coin (19) (i.e., the covariates have no predictive power). The AU-ROC of the Step 1 model quantifies the accuracy of using individual-level information alone for identifying individuals with the outcome.

Step 2 – The general contextual effects model: Step 2 consists of extending the Step 1 model from a conventional single-level logistic regression model to a two-level individuals-within-neighbourhoods logistic regression model. This extended model is written as

$$y_{ij} \sim \text{Binomial}(1, \pi_{ij}), \qquad (2)$$

$$\text{logit}(\pi_{ij}) = \beta_0 + \beta_1\texttt{age}_{ij} + \beta_2\texttt{sex}_{ij} + \beta_3\texttt{income}_{ij} + u_j,$$

$$u_j \sim N(0, \sigma_u^2),$$

where u_j denotes the random effect for neighbourhood j. These effects are assumed normally distributed with zero mean and variance σ_u^2, a parameter to be estimated.

Postestimation, values can be assigned to these effects via empirical Bayes prediction. These predictions $\hat{u}_j$ are sometimes referred to as shrinkage estimates as their values are shrunk towards the population-average of zero by a shrinkage factor proportional to the amount of information available on each neighbourhood (essentially the neighbourhood size). Shrinkage is desirable as it protects one against over interpreting otherwise extreme predictions typically associated with very small neighbourhoods. The statistical uncertainty surrounding these predictions can also be calculated and communicated via error bars (e.g., 95% confidence intervals). This uncertainty must be taken into account when ranking neighbourhoods, for example by predicted prevalence of the health outcome, as such rankings have been shown to be especially unreliable (see elsewhere for an extended explanation and empirical examples) (37-39). More generally, the interpretation of neighbourhood rankings needs be done in relation to the *general contextual effect* (see elsewhere for empirical examples)(2).

The *general contextual effect* is appraised using the estimated between-neighbourhood variance $\hat{\sigma}_u^2$ as this quantifies the variability in unobserved influences on the health outcome common to individuals living in in the same neighbourhood. Thus, $\hat{\sigma}_u^2$ is assumed to reflect variation in any direct effects of neighbourhood context captured by the neighbourhood boundaries (i.e., “causal” effect of place). However, in an observational study, it might also reflect neighbourhood compositional differences in unmodelled individual characteristics (e.g., unobserved selection of individuals into neighbourhoods). We calculated three different measures of *general contextual effects*: (i) the change in the AU-ROC compared with the Step 1 model; (ii) the ICC; and (iii) the MOR.

(i) While the AU-ROC of the Step 1 model quantifies the accuracy of using individual-level information alone for identifying individuals with, or without the outcome, the predicted probabilities from the Step 2 model are based on both the individual-level covariates and the predicted neighbourhood random effect $\hat{u}_j$. Consequently, the AU-ROC of the Step 2 model can be compared with that from Step 1 to quantify the added value of having information on the neighbourhood of one's residence when it comes to identifying the outcome of the individuals. Therefore, in this approach the general contextual effect of the neighbourhood is appraised by quantifying the increase in the AU-ROC achieved when adding general neighbourhood information to the individual level predictions calculated in the Step 1 model. The larger this difference, the greater the general neighbourhood effect is.

(ii) We chose to calculate the ICC based on the latent response formulation of the model as it is the approach most widely adopted in applied work. This formulation assumes a latent continuous response underlies the observed binary response and it is this latent response for which the ICC is calculated and interpreted. The higher the ICC, the more relevant neighbourhood context is for understanding individual latent response variation (10, 12, 25). The ICC is calculated as

$$\rho = \frac{\sigma_u^2}{\sigma_u^2 + \frac{\pi^2}{3}}$$

where $\frac{\pi^2}{3}$ denotes the variance of a standard logistic distribution. (Note that here π denotes the mathematical constant 3.1416…, not the probability.)

(iii) The MOR (10, 28, 29) is an alternative way of interpreting the magnitude of the neighbourhood variance. The MOR translates the neighbourhood variance estimated on the log-odds scale, to the widely used OR scale. This makes the MOR comparable with the OR of individual and neighbourhood covariates. The MOR is defined as the median value of the

distribution of ORs obtained when randomly picking two individuals with the same covariate values from two different neighbourhoods, and comparing the one from the higher risk neighbourhood to the one from the lower risk neighbourhood. In simple terms, the MOR can be interpreted as the median increased odds of reporting the outcome if an individual moves to another neighbourhood with higher risk. Therefore, the higher the MOR the greater the general contextual effect. The MOR is calculated as

$$\text{MOR} = \exp\left(\sqrt{2\sigma_u^2}\Phi^{-1}(0.75)\right),$$

where $\Phi^{-1}(\cdot)$ represents the inverse cumulative standard normal distribution function. In absence of neighbourhood variation (i.e., $\sigma_u^2 = 0$), the MOR is equal to 1.

Step 3 – The specific contextual effects model: Step 3 consists of adding the neighbourhood covariate of interest to the model in order to estimate the specific OR for a contextual variable. In our case we are interested in the effect of neighbourhood income (i.e., rich or poor) on each outcome. The step 3 model can be written as

$$y_{ij} \sim \text{Binomial}(1, \pi_{ij}), \tag{3}$$

$$\text{logit}(\pi_{ij}) = \beta_0 + \beta_1 \texttt{age}_{ij} + \beta_2 \texttt{sex}_{ij} + \beta_3 \texttt{income}_{ij} + \beta_4 \texttt{nincome}_j + u_j,$$

$$u_j \sim N(0, \sigma_u^2),$$

where `nincome` denotes the additional neighbourhood covariate.

Specific contextual effects measure the associations between contextual characteristics of the neighbourhood (e.g., rich or poor neighbourhood) and the individual outcome. As in the case of individual-level observational effects, specific contextual effects are estimated using measures of average effect such as ORs. However, an extended misunderstanding when applying multilevel regression analyses is to give a “population average” interpretation to the OR of contextual variables (10, 28, 29).

The point is that the multilevel regression provides regression coefficients for individual variables that are adjusted for the neighbourhood-level random effects. That is, they reflect the association between individual level variables and the outcome within a specific neighbourhood. They are therefore termed ‘‘neighbourhood specific” or ‘‘cluster specific” ORs. However, in multilevel logistic regression, a contextual OR can hardly be interpreted in this way since the contextual variable is constant for all individuals in the neighbourhood. The contextual OR can at best be interpreted as contrasting two neighbourhoods differing in the value of the contextual variable by one-unit, but which have identical value for the neighbourhood-level random effects (and all other covariates). To avoid this difficult interpretation, Larsen *et al* (28, 29) proposed the use of the IOR-80% as a way of including the neighborhood variance in the quantification of a contextual OR.

The lower and upper bounds of the IOR-80% for `nincome` are calculated as

$$\exp\left(\beta_4 \pm \sqrt{2\sigma_u^2}\ \Phi^{-1}(0.9)\right).$$

The IOR-80% is defined as the middle 80% range of the distribution of ORs formed by making random pairwise comparison between neighbourhoods exposed and non-exposed to the contextual variable. The IOR-80% interval is narrow if the between-neighbourhood variance σ_u^2 is small, and it is wide if the between-neighbourhood variance is large. If the IOR-80% interval contains 1, then for some neighbourhoods the association is in the opposite direction to the overall OR (28) (10).

An alternative to the IOR-80% is the Proportion of Opposed Odds Ratios (POOR). That is, the proportion of ORs with the opposite direction to the overall OR (10). The values of the POOR extend between 0% and 50%. A POOR of 0% means all ORs have the same sign. A POOR of 50% means that half of the ORs are of the opposite sign and so the association is

very heterogeneous. For our binary measure of neighbourhood income, the POOR is calculated as

$$\text{POOR} = \Phi\left(-\frac{\beta_4}{\sqrt{2\sigma_u^2}}\right).$$

Observe that in Step 2 we calculated the AU-ROC as a way of quantifying neighbourhood general contextual effects. In Step 3, we included a specific contextual characteristic of the neighbourhood (i.e., low neighbourhood income) into the model in order to quantify specific contextual effects. However, adding this specific contextual variable cannot increase the AU-ROC obtained in the Step 2 model since that model gives the maximum AU-ROC that can be obtained by combining the available individual information and the neighbourhood identity. The latter captures the totality of potentially observable, but also unobservable neighbourhood factors. The inclusion of a specific neighbourhood contextual variable as a fixed-effect covariate will explain some of that neighbourhood variance (that is, decrease the average absolute size of the neighbourhood u_j estimates) and, thereby reducing the predictive role of the neighbourhood random effects. However, this change to the model specification simultaneously improves the model prediction through the addition of the regression coefficient for the neighbourhood income variable. Because of this balance the discriminatory accuracy of the Step 2 and 3 models will be effectively the same.

Step 3 provides a way of understanding the mechanism behind the observed general contextual effects. For this purpose we can calculate the proportional change in variance (PCV) defined as the proportion of the neighbourhood variance in Model 2 explained by adding the specific neighbourhood effect (i.e., neighbourhood income variable) in Model 3

$$\text{PCV} = \frac{\sigma^2_{u[\text{Model 2}]} - \sigma^2_{u[\text{Model 3}]}}{\sigma^2_{u[\text{Model 2}]}}.$$

In our case, a large PCV would suggest that the general contextual effect is substantially mediated by the neighbourhood income variable.

Summary of the multilevel analysis of heterogeneity approach

In multilevel analysis of heterogeneity, we need a joined analysis that includes individual variables, neighbourhood boundaries, and neighbourhood characteristics. We need to include measures of association, variance and discriminatory accuracy. The simplistic "risk factor" approach based on the calculation of ORs alone is insufficient

In our two example studies we perform a series of three consecutive regression models.

We start with Model 1 (Step 1) that only includes individual-level covariates in a standard (i.e., single-level) logistic regression. The selection of these individual variables is based on the assumption that they condition the outcome and also the neighbourhood of residence. For instance, age is associated with use of psychotropic medicine and individuals may move to certain neighbourhoods when they become older. That is, we aim to prevent compositional confounding in later regression analyses. The candidate individual-level variables are not mediators of the neighbourhood effects. In our example the neighbourhood cannot change the age of the individuals. Besides the average ORs for the individual-level variables, the fundamental information in Model 1 is the size of the AU-ROC.

In Model 2 (Step 2) we quantify the added value of having neighbourhood level information. We only include the neighbourhood boundaries without specifying any neighbourhood characteristic. We analyse the change in the AU-ROC compared with Model 1. We also interpret the ICC and the MOR. This information tells us about the size of the general contextual effect.

In the final model, Model 3 (Step 3), we include specific neighbourhood information (neighbourhood income). In this model, the interpretation of the OR, the IOR and POOR must

always be done in relation to the neighbourhood variance σ_u^2 of Model 2 and the PCV associated with moving from Model 2 to Model 3. For instance, suppose Model 2 estimated a high value for σ_u^2 and therefore a high ICC for the binary outcome "visiting a private vs. a public GP". Thereafter, in Model 3, we include a contextual variable (neighbourhood high income). If neighbourhood high income is associated with the outcome (a high OR) and it explains a large share of σ_u^2 (PCV is high) the IOR-80% will be narrow and the POOR low. This case illustrates a situation where the neighbourhood context conditions the outcome (i.e., high σ_u^2 and ICC). It also demonstrates that this influence appears mediated by the contextual variable (neighbourhood high income) so the contextual variable is not only strongly associated with the outcome but it also explains the neighbourhood variance and thereby shows a narrow IOR-80% or a low POOR. In other words, the conclusion would be that the neighbourhood context influences the individual choice of GP and that this influence has to do with the socioeconomic circumstances of the neighbourhoods

However, there are other possible situations. For instance, σ_u^2 could be very low from the beginning (Model 2) and the contextual variable could be significantly associated with the outcome but still does not explain much of the σ_u^2 (i.e., low PCV) in Model 3. Nevertheless, since σ_u^2 was low from the beginning, the IOR-80% would also be narrow and the POOR low. In this case the neighbourhood context would have a small influence on the individual choice of GP even if the socioeconomic circumstances of the neighbourhoods are, on average, associated with the outcome and the IOR-80% is narrow.

Model estimation

The models were estimated using Markov chain Monte Carlo (MCMC) methods as implemented in the MLwiN multilevel modelling software (40). We specify diffuse (vague, flat, or minimally informative) prior distributions for all parameters. We use quasilikelihood estimation to provide good starting values for all parameters. For each model, we specified a

burn-in length of 5,000 iterations and a monitoring chain length of 10,000 iterations. Visual assessments of the parameter chains and standard MCMC convergence diagnostics suggest that the lengths of these periods are sufficient. The Bayesian deviance information criterion (DIC) was used as a measure of goodness of fit of our models (41). The DIC considers both the model deviance and its complexity. Models with smaller DIC are preferred to models with larger DIC, with differences of five or more considered substantial (42).

Online supplementary materials

A fully anonymized version of the data is provided in the Online Supplementary Materials. We also provide the saved MLwiN worksheet for each model and an Excel sheet for the calculation of the ICC, MOR, 80%IOR and the POOR. A Stata do-file and dataset is also made available for users of that software.

Ethics statement

The National Board of Health and Welfare and Statistics Sweden constructed the database by means of record linkage of different registers using the unique Swedish personal identification number. Finally, the Swedish authorities delivered the research database to us without the personal identification numbers to ensure the anonymity of the subjects. The Regional Ethics Review Board in southern Sweden as well as the data safety committees from the National Board of Health and Welfare and from Statistics Sweden approved the construction of the LOMAS database.

For the purpose of our study we created a fully anonymized sample that completely prevents the identification of individuals using a combination of variables. This fully anonymized sample is provided in the Online Supplementary Materials.

Results

Characteristics of the population (Table 1)

In the study sample, use of psychotropic drugs was more frequent in individuals with low income and in poor neighbourhoods while the opposite was true for visiting a private GP. Rich neighbourhoods had a higher percentage of people 55 years or older and a slightly lower percentage of men than poor neighbourhoods.

Table 1. Characteristics of the population 35 – 65 year-olds in Malmö, 2006 by neighbourhood income

	Neighbourhood income	
	Poor (N= 93)	Rich (N= 125)
Number of individuals	22780	20511
Psychotropic drugs	29%	23%
Private GP	11%	35%
Low income	60%	27%
Men	45%	42%
Age (year-groups)		
35 – 39	19%	17%
40 – 44	18%	17%
45 – 49	17%	15%
50 – 54	16%	16%
55 – 59	16%	18%
60 – 64	14%	17%

Analysis of the use of psychotropic drugs (Table 2)

Specific Individual Average Observational Effects

The individual level population average Model 1 shows that use of psychotropic drugs increases monotonically with age and was more frequent for women and among people with low income. These individual characteristics, however, were not sufficient for predicting individuals' use of psychotropic drugs with any degree of accuracy since the AU-ROC was low (i.e., 0.616.) (Figure 1). In Model 2, the cluster specific association between individual income and use of psychotropic drugs was lower than the population average association in Model 1.

Table 2. Multilevel logistic regression analysis of psychotropic drug use in the 35 – 65 year-old population of Malmö, 2006. Values are odds ratios (OR) with 95% confidence interval (CI) unless stated otherwise. The intercept is not shown in the table.

	Simple logistic regression analysis	Multilevel logistic regression analysis	
	Model 1	Model 2	Model 3
**Specific Individual Average Effects**			
Men vs. women	0.61 (0.58 – 0.64)	0.60 (0.58 – 0.63)	0.60 (0.58 – 0.63)
Age groups			
35 – 39	Reference		
40 – 44	1.35 (1.24 – 1.46)	1.35 (1.25 – 1.45)	1.35 (1.24 – 1.46)
45 – 49	1.63 (1.50 – 1.77)	1.64 (1.51 – 1.77)	1.63 (1.51 – 1.77)
50 – 54	1.81 (1.67 – 1.95)	1.82 (1.69 – 1.96)	1.82 (1.68 – 1.97)
55 – 59	1.91 (1.76 – 2.07)	1.94 (1.80 – 2.10)	1.95 (1.81 – 2.10)
60 – 64	1.95 (1.80 – 2 .11)	2.00 (1.85 – 2.16)	2.01 (1.86 – 2.17)
Low vs. high income	1.67 (1.60 – 1.74)	1.56 (1.49 – 1.64)	1.52 (1.44 – 1.59)
**Specific Contextual Average Effects**			
Low vs. high neigh income			1.29 (1.21 – 1.38)
80% IOR			0.99 – 1.69
POOR (%)			11
**General contextual effects**			
Neighbourhood variance		0.038 (0.026 – 0.054)	0.022 (0.012 – 0.035)
PCV (%)			42
ICC (%)		1.1 (0.8 – 1.6)	0.7 (0.4 – 1.1)
MOR		1.20 (1.17 – 1.25)	1.16 (1.11 – 1.20)
AU-ROC	0.616 (0.610 – 0.622)	0.630 (0.625 – 0.636)	0.629 (0.623 – 0.635)
AU-ROC change*		0.014	-0.001?
**Goodness of fit**			
DIC	48205	48063	48041
DIC change*		-142	-22

IOR: interval odds ratio. POOR: proportion of opposed odds ratios. PCV: proportional change in the variance. ICC: intra-class correlation coefficient. MOR: median odds ratio. AU-ROC: area under the receiver operating characteristic curve. DIC: Bayesian deviance information criterion. *: Change in relation to the previous model.

Specific Contextual Average Observational Effects: IOR and POOR

In Model 3 we observed that, over and above individual income, age and sex, living in a low income neighbourhood conclusively increased the individual probability of use of psychotropic drugs (i.e., OR= 1.29). However, the 80%-IOR included 1 and the percentage of ORs of opposite direction was considerable (POOR=11%).

General Contextual Observational Effects: neighbourhood variance, ICC, MOR and AU-ROC

In Model 2, The ICC and the MOR were low (i.e., 1.1% and 1.20 respectively) which indicated that the neighbourhoods, as defined by the SAMS geographical boundaries, do not appear to capture a relevant context for understanding an individual's propensity of using psychotropic drugs.

The added value of knowing an individual's neighbourhood of residence besides individual information (age, sex and income) was very small since the AU-ROC only increased 0.014 units when comparing Model 2 with Model 1 (Fig. 1).

In Model 3, inclusion of the neighbourhood income variable explained 42% of the neighbourhood variance and decreased the ICC and MOR values to 0.7% and 1.16 respectively.

Figure 1: Areas under the receiver operating characteristic (AU-ROC) curve for use of psychotropic drugs during 2006 in the city of Malmö, Sweden plotted separately for Model 1 which only adjusts for individual-level covariates age, sex and income (black thick line), and Model 2 which additionally adjust for neighbourhood of residence (grey dotted line)

Figure 2 shows the ranking of the neighbourhoods of Malmö in 2006 according to their logarithmic (log) odds ratio (OR) of using psychotropic drugs, having the average of the whole city sample as reference. Fig. 2A represents the values obtained from a model including age, sex and individual income (Model 2); and Fig. 2B represents a model which additionally adjusts for neighbourhood income (Model 3).

Figure 2: Ranking of the neighbourhoods of Malmö in 2006 according their use of psychotropic drugs. The values are obtained from multilevel logistic regression analyses and represent logarithmic (log) odds ratio (OR), having the average of the whole city sample as reference. (A) Represents the values obtained from Model 1 which includes age,

sex and individual income; and (B) Model 2 which additionally adjusts for neighbourhood income. The value of the intra-class correlation coefficient (ICC) is included as a percentage.

Figure 2 indicates that there was considerable uncertainty in the ranking of the neighbourhoods, which expressed itself as a substantial overlapping of the confidence intervals. These "league tables" are only based on neighbourhood differences and need to be interpreted side-by-side with measures of general neighbourhood effects. Indeed, the ICC was very low in both models.

Analysis of choosing a private vs. a public specialist physician in general practice (Table 3)

Specific Individual Average Observational Effects

The population average Model 1 indicates that the odds of choosing a private GP were similar for men and women, and that they were somewhat higher among individuals aged 50 to 64 than among younger individuals. High individual income clearly increased the odds of choosing a private GP. These individual characteristics, however, were not sufficient for predicting individuals' choice of GP with any degree of accuracy since the Model 1 AU-ROC was low (i.e., 0.600)

Interestingly, the association between individual income and choosing a private GP declined when we recognized the multilevel structure of the data and included the neighbourhood level as a random effect in Model 2. This situation expresses the fact that the individual association in Model 1 was capturing not only a modest within neighbourhood association but also a stronger between neighbourhood association, A situation that was confirmed in Model 3 (see under "Specific contextual average effects") since the neighbourhood income was, on average, strongly associated to choosing a private GP.

Specific contextual effects: IOR and POOR

Model 3 shows that high neighbourhood income was, on average, strongly associated with visiting a private physician (OR= 3.50). So the customary interpretation would be that, over and above individual income, age and sex, living in a high income neighbourhood strongly increased the individual probability of visiting a private physician. However, this contextual variable only explained a small share (PCV= 11%) of the initially large neighbourhood variance. Therefore, unmodeled variability between neighbourhoods remained large as expressed by the wide IOR-80% = 130.28 - 0.09. Also the POOR indicated that 33% of the time an individual from a high income neighbourhood had a lower, rather that higher, likelihood of visiting a private GP than an individual from a low income neighbourhood. That is, the average OR hides strong heterogeneity around the average association.

General Contextual Effects: Neighbourhood variance, ICC, MOR and AU-ROC

If the neighbourhood context were relevant for understanding individuals' choice of private vs public GPs we would expect a high ICC, a high MOR and a high increase of the AU-ROC in Model 2 compared to Model 1. This is just what we found. The ICC in Model 2 was close to 60% and the MOR close to 8 which are very high values in "neighbourhood and health" studies. Furthermore, adding information on neighbourhood in Model 2 increased the AU-ROC of Model 1 from about 0.6 to almost 0.9 which indicates that knowing the neighbourhood of one's residence allows us to predict with rather high accuracy if an individual will choose a private versus a public GP (see Figure 3).

If the large observed general neighbourhood effect were mediated by neighbourhood income (or by other unobserved neighbourhood characteristics that this covariate may proxy for) we would expect a considerable reduction of the neighbourhood variance, the ICC, and the MOR in Model 3 compared with Model 2. However, this was not the case. In support of this argument, measuring the AU-ROC using only individual variables and neighbourhood income but not the neighbourhood random effect gave an AU-ROC (95% confidence interval) =

0.620 (0.614 – 0.626) which is only 0.03 units higher that Model 1 with only individual level variables.

Table 3. Multilevel logistic regression analysis of choosing a private versus a public specialist in the 35 – 65 year-old population of Malmö, 2006, Values are odds ratios (OR) and 95% confidence interval (CI) unless stated otherwise.

	Simple logistic regression analysis	Multilevel logistic regression analysis	
	Model 1	Model 2	Model 3
Specific individual average effects			
Men vs. women	0.96 (0.92 – 1.01)	0.94 (0.88 – 1.01)	0.94 (0.88 – 1.01)
Age groups			
35 – 39	Reference		
40 – 44	1.01 (0.93 – 1.09)	1.07 (0.94 – 1.20)	1.07 (0.94 – 1.20)
45 – 49	1.02 (0.94 – 1.11)	1.22 (1.07 – 1.37)	1.22 (1.07 – 1.37)
50 – 54	1.08 (1.00 – 1.17)	1.25 (1.10 – 1.41)	1.25 (1.10 – 1.41)
55 – 59	1.21 (1.12 – 1.31)	1.30 (1.16 – 1.46)	1.30 (1.16 – 1.46)
60 – 64	1.20 (1.10 – 1.30)	1.20 (1.06 – 1.35)	1.20 (1.06 – 1.35)
High vs. low income	2.13 (2.02 – 2.24)	1.14 (1.06 – 1.22)	1.14 (1.06 – 1.22)
Specific contextual average effects			
High vs. low neighbourhood income			3.50 (2.13 – 5.78)
80% IOR			0.09 – 130.28
POOR (%)			33
General contextual effects*			
Neighbourhood variance		4.479 (3.699 – 5.502)	3.980 (3.277 – 4.892)
PCV (%)			11
ICC (%)		57.8 (53.1 – 62.7)	54.9 (50.1 – 59.9)
MOR		7.53 (6.42 – 9.37)	6.71 (5.62 – 8.25)
AU-ROC	0.600 (0.593 – 0.606)	0.895 (0.891 – 0.899)	0.895 (0.891 – 0.899)
AU-ROC change*		0.295	0.000
Goodness of fit			
DIC	44726	24647	24648
DIC change*		-20079	1.28

IOR: interval odds ratio. POOR: proportion of opposed odds ratios. PCV: proportional change in the variance. ICC: intra-class correlation coefficient. MOR: median odds ratio. AU-ROC: area under the receiver operating characteristic curve. DIC: Bayesian diagnostic information criterion.*: change in relation to the previous model

Figure 3. Areas under the receiver operating characteristic curve (AU-ROC) for choosing a private vs. a public GP during 2006 in the city of Malmö, Sweden plotted separately for Model 1 which only adjusts for individual-level covariates age, gender and income (black thick line); and for Model 2 which additionally adjusts for the neighbourhood of residence (grey dotted line)

Fig, 4 shows the ranking of the neighbourhoods of Malmö in 2006 according to their log OR of visiting a private GP, having the average of the whole city sample as reference. Fig. 4A represents the values obtained from a model including age, sex and individual income (Model 2); and Fig. 4B represents a model which additionally adjusts for neighbourhood income (Model 3).

Figure 4: Ranking of the neighbourhoods of Malmö in 2006 according their use of a private GP. The values are obtained from multilevel logistic regression analyses and represent logarithmic (log) odds ratio (OR), having the average of the whole city sample as reference. (A) represents the log ORs obtained from Model 1 which includes age, sex and individual income; and (B) Model 2 which additionally adjusts for neighbourhood income. The value of the intra-class correlation (ICC) coefficient is included as a percentage

We observed a bimodal distribution for the neighbourhood differences with two groups of neighbourhoods, one smaller group with a higher probability of visiting a private GP, and another larger group with a lower probability. This bimodality reflects the underlying nature of private GP use. In our case, it revealed that over and above age, sex and individual income, individuals in some neighbourhoods mostly visit private physicians while individuals in other neighbourhoods mostly visit public GPs. A similar bimodality is frequently observed when there are strong general contextual effects as is the case when analysing individual within households (2, 43), sibling within families (44), or children within mothers (45).

This bimodality was not a concern for the statistical analysis as the number of neighbourhoods was high, which makes the assumption of normally distributed random

effects less relevant (46). Nevertheless, adjusting for neighbourhood income (low vs high) reduced the bimodality and it is assumable that the bimodality might be further reduced by modelling neighbourhood income in a more flexibly way (e.g., by entering a continuous measure of income as a polynomial). The pattern of neighbourhood differences also suggests the existence of spatial correlation which could be conditioned by the segregation of private practices in specific geographical areas. It is possible to allow for spatially correlated random effects in multilevel logistic regression, but this is beyond the scope of the current article.

We also note that there was high individual socioeconomic segregation. Multilevel logistic regression analyses have recently been proposed for modelling social and other forms of segregation (47-49). Applying those ideas to our data, we fit a separate multilevel logistic regression analyses, modelling low individual income as the response variable. We estimated a neighbourhood variance of 1.032 which corresponds to an ICC of 24% and substantial segregation. Therefore, adjusting neighbourhood income for individual income is based on strong extrapolations since there are few individuals with high income living in poor neighbourhoods as well as few individuals with low income living in rich neighbourhoods.

Discussion

We have presented two applications illustrating how to use multilevel logistic regression analysis of heterogeneity to estimate individual and neighbourhood influences on individual health and health care utilization. Our three-step approach distinguishes between specific (measures of association) and general (measures of variance) contextual effects, and demonstrates the relevance of combining both approaches for gaining greater substantive understanding of the phenomenon under study. We analyse two different individual outcomes (psychotropic drug use and visit to a private vs. public GP) for which the relative importance of neighbourhood influences differs substantially. Our results agree with previous studies on

the city of Malmö observing a large general neighbourhood effect for individual choice of private physician in 1999 (i.e., ICC = 33%, MOR= 3.36) (28) but a minor general neighbourhood effect for use of anxiolytic-hypnotic drugs (i.e., ICC= 1.7%, MOR = 1.25) in 1991-1996 (50).

We question the current probabilistic, risk factor epidemiological approach based on the simple interpretation of ORs for specific individual and contextual (e.g., neighbourhood) characteristics in isolation (18). We promote a three-step multilevel analytical approach. Step 1 consists of fitting a single-level logistic regression adjusting for only the individual-level covariates, then evaluating the ORs and calculating the discriminatory accuracy (e.g., AU-ROC) of these variables. Step 2 consists of extending the model to two-levels (by adding the neighbourhood random effect) and then assessing the importance of general contextual effects using the ICC and AU-ROC. Step 3 consists of adding specific neighbourhood characteristics (i.e., specific neighbourhood effects) to the model and interpreting their ORs jointly with the size of the initial general contextual effect and the size of the neighbourhood variance explained (i.e., PCV). We argue that the incorrect population average interpretation of the OR for contextual variables needs be avoided. For this purpose the IOR or the POOR should be presented side-by-side with the average OR.

Psychotropic drug use

Applying our three-step approach to psychotropic drug use, we observed that sex, increased age, and individual low income were associated with the use of this medication. However, the information provided by these individual characteristics did not allow users of psychotropic drugs to be distinguished from non-users with any degree of accuracy (AU-ROC= 0.616). We also observed a very small general contextual effect since accounting for neighbourhood of residence only increased the AU-ROC by 0.014 units and both the ICC (i.e., 1.1%) and the

MOR (i.e., 1.20) were very low. In fact, our results suggest that SAMS neighbourhoods were more similar to simple random samples from the population of Malmö, than to meaningful contexts influencing individual psychotropic drug use.

The low AU-ROC of the neighbourhood context (i.e., the low general contextual effects) needs to be considered when interpreting the small but conclusive association between low neighbourhood income and individual use of psychotropic drugs. One could argue that this neighbourhood variable explained 42% of the neighbourhood variance, but as such variance was rather small (i.e., $\hat{\sigma}_u^2$ = 0.038), it actually explained a lot of very little. Furthermore, the POOR informed that 11% of the time the positive association between low neighbourhood income and individual psychotropic drug use was in the opposite direction with a decreased, rather than increased, propensity of using psychotropic drugs in the low income neighbourhoods.

Paradoxically, when the neighbourhood variance is low (i.e., there is a weak general contextual effect) it is easier to obtain “significant” associations with narrow 95% CI for the contextual variables (i.e., specific contextual effect). This situation happens because we assign the values of neighbourhood variable to uncorrelated individuals in the sample. In other words, the less neighbourhood boundaries matter for the outcome, the easier it is to get “significant” associations between specific neighbourhood characteristics and the individual outcome. When researchers plan a study of “neighbourhood and health”, they typically assume that there is a strong intra-neighbourhood correlation. However, we need to check this assumption and always interpreted the specific contextual effect (i.e., OR and 95% confidence interval) considering the size of the initial general contextual effects (e.g., ICC or AU-ROC). Following the three-stage approach promoted in this article ensures a more appropriate interpretation.

The low general neighbourhood effects could be related to the fact that psychotropic drug use may be conditioned by other kind of contexts like the physicians or the Primary Health Care centres where the individuals are treated. The SAMS areas were relatively easy to obtain but their definition was not based on robust theory related to the contextual processes and mechanisms that may condition use of psychotropic drugs (or, for that matter, the choice of a private GP). In fact, the relevant context may not be at the neighbourhood level at all. Prescription of psychotropic drugs is homogenously regulated all over Sweden (51), which may reduce the influence of the neighbourhood on individual use of this medication. However, larger contextual effects might be observed when studying countries with different health care systems and therapeutic traditions or where psychotropic drugs are available over the counter. We have previously observed such a situation in the context of studying blood pressure. We identified a very low general contextual effect of the city areas in Malmö (6), but this effect was much higher when analysing countries with different health care systems (7)

In summary, we were not able to identify with accuracy the factors that predict psychotropic drug use. What we did find was that individual age, sex, and low income appeared to be poor predictors for identifying users of psychotropic drugs, and additionally including neighbourhood of residence did not alter this situation. That is, the neighbourhood context had only a negligible influence on individual use of psychotropic drugs.

Choice of a private vs. a public GP

Concerning individual choice of private vs. public GP, our analysis showed that while the sex of the individual was not related to this choice, age was weakly positively associated and individual high income strongly associated (OR = 2.13) to this choice. However, as in the case of psychotropic drug use, the low discriminatory accuracy (AU-ROC= 0.600) rendered

the information supplied by these individual-level covariates insufficient for distinguishing who would choose a private vs. public GP. However, we found a very strong general contextual effect since accounting for neighbourhood of residence in the analysis increased by 0.295 units the AU-ROC to 0.895. Also, the large ICC (i.e., 57.8%) and MOR (i.e., 7.53) values indicate that SAMS neighbourhoods captured a meaningful context influencing this individual behaviour. The socioeconomic context of the neighbourhoods (i.e., high vs. low neighbourhood income) was, on average, associated with choosing a private GP (OR = 3.50). However, this specific neighbourhood variable only explained 11% of the large neighbourhood variance in Model 2 (i.e., $\hat{\sigma}_u^2$ = 4.479). In fact, in as much as 33% of comparisons between rich and poor neighbourhoods, the OR for neighbourhood income was in the opposite direction so high neighbourhood income was associated to a lower rather than a higher propensity of choosing a private GP.

We observed that, on average, utilization of private GPs was higher among high income people and in high income neighbourhoods than in the low income categories, which deserves a closer analysis. In fact, access to health care in Sweden is by law (52) on equal terms and according to needs, and for many years societal funding has equally financed both public and private health (53) so economic circumstances should not be the main reason for choosing a public vs. a private GP (53). The observed link between income and utilization of private GPs might depend on cultural preferences rather than solely on economic reasons. It is known, for example, that choice of sector also carries a symbolic meaning (54) and high income individuals have been argued to intrinsically prefer private care. However, an alternative explanation could be the existence of “invisible” barriers like adverse attitudes of private GPs against low income individuals, which might channel those individuals towards public GPs (53).

In summary, over and above individual characteristics the neighbourhood of residence strongly predicted the choice of a private vs. a public GP, but the reasons for this phenomenon are only partially explained by socioeconomic circumstances of the neighbourhoods. On average, individuals residing in high income neighbourhoods had a higher propensity of visiting a private GP, but this contextual variable only explained a low proportion of the variation in neighbourhood differences. Other contextual factors not considered in our analysis, for instance, the degree of private GP provision in each neighbourhood might go some way to explaining the observed general contextual effects.

Public Health implications

Our results are relevant when planning public health interventions. For example, policies to improve psychological health or reduce the use of psychotropic drugs in the city of Malmö, would need to realize that focusing on specific neighbourhoods would not be effective because of the low discriminatory accuracy of this information. In fact, the same is true for the individual characteristics we analysed: age, sex, and income. Put differently, neither neighbourhood of residence nor the individual characteristics studied provided accurate information for identifying target groups. If policy makers do choose to focus on those individuals and neighbourhood with a higher average risk of using psychotropic drugs (which would be the normal procedure in risk factors epidemiology), they need to be aware that many psychotropic users would be labelled as “low risk” and that many non-users of psychotropic drugs would be labelled as “high-risk”. That is, focusing on only high risk groups would unnecessarily expose many individuals to an intervention they do not need and would leave many individuals untreated because they belong to low risk groups. Perhaps a better approach would be to launch an intervention on the whole population. In any case, considering the balance between harms and benefits, an intervention with low discriminatory accuracy conveys that the principle of *primum non nocere* must be an absolute condition.

The public health implications of our second analysis are very different. Here, policies to increase the use of public GP services should mostly focus on specific neighbourhoods, perhaps by opening local public GP alternatives.

Multilevel analysis of heterogeneity and risk factors epidemiology

The multilevel analysis of heterogeneity we present in our study is rather innovative (18). Most studies analysing the role of individual or contextual variables on health adopt a probabilistic perspective based on the analysis of differences in average risk between exposed and unexposed groups (55) but without recognizing the value of analysing variance (56) . This is the classical approach in so called "risk factors epidemiology" and many multilevel analyses have only focused on the identification of contextual risk factors such as neighbourhood social capital and neighbourhood deprivation. From this perspective small or even tiny effects (e.g., OR = 1.5 or even lower) with very low discriminatory accuracy are considered relevant. The problem is that by doing so we promote population level policies and interventions that may lead to both under and overtreatment, as well as unnecessary side effects and costs. It also raises ethical concerns related to misleading risk communication and the perils of both unwarranted interventions and stigmatization of exposed individuals (57).

The multilevel analytical approach we propose differs fundamentally from the classical one. First, we adopt a mechanistic perspective that tries to understand the individual heterogeneity of responses surrounding average probabilities. Second, we combine measures of association with measures of variance and discriminatory accuracy and stress the importance of evaluating not only the discriminatory accuracy of the individual level variables but also of the geographical boundaries used to define neighbourhoods in relation to the outcome under investigation. For this purpose what we denominated *general contextual effects* in multilevel regression analysis allows us to quantify the degree of clustering within neighbourhoods (i.e.,

the ICC) (3, 10) or, analogously, the discriminatory accuracy of using the boundaries of the neighbourhoods in the analysis (i.e., the AU-ROC) (32, 33). The existence of individual dependence within neighbourhoods is not only the *sine qua non* for applying statistical multilevel analyses but also the size of this dependence provides fundamental substantive information (1, 18).

Strength and weaknesses

Our current study tries to quantify the relevance of neighbourhoods in Malmö for understanding individual use of psychotropic drugs and choice of private vs public GP. We considered the simplest possible multilevel structure of individual nested within neighbourhoods as this is the most common design in neighbourhood and health studies. However, to constrain the study of contextual effects to a single geographical level (e.g., SAMS areas) is certainly an extreme simplification (58). Individuals are likely to be simultaneously affected by multiple contexts at different scales across time (59-63). Nevertheless, the analytical approach we promote can be developed for more than two levels of analyses (e.g., individuals nested in households nested in neighbourhoods)(2) as well as for multiple membership and cross-classified multilevel structures (e.g., schools and neighbourhoods at different times in the life course) (18, 60, 64-66). However, adopting a pragmatic rather than academic perspective, straightforward multilevel analysis of heterogeneity that only considers individuals nested in neighbourhoods provides a better basis for informed decisions in public health than the simple ecological or spatial analyses of small area variation or classical multilevel analysis of contextual risk factors (2).

The identification of causal effects in observational epidemiology and, more specifically, in the study of neighbourhoods “effects” is a major problem. In our study, the underlying causal question was to know what would happen to an individual if she/he, *ceteris paribus*, moves to

another neighbourhood with a different context. Furthermore, we wanted to identify if any general effect was mediated by a specific variable informing the socioeconomic characteristics of the context (e.g., rich vs. poor neighbourhood). However, what "rich" and "poor" neighbourhood means is difficult to specify and it would need a deeper sociological analysis. In the adjusted analysis we only considered individual age, sex and income as our main purpose was to illustrate the methodology. Therefore, we cannot exclude the existence of omitted confounding factors. Nevertheless, in neighbourhood analyses it is always a caveat to distinguish between confounder and mediator variables as frequently a common cause of both place of residence and the health outcome may also be a mediator of the neighbourhood effect (for instance low income is associated to using psychotropic drugs and low income individuals may be segregated to poor neighbourhoods but, in turn, living in a poor neighbourhood may reduce the chances of increasing an individual's income). Furthermore, there may be problems of extrapolation (i.e., making inferences beyond the range of the data,) since few rich individuals reside in poor neighbourhoods and vice versa, so the appropriateness of adjusting for individual income could be questioned. Finally, while some contextual effects may be caused by exogenous exposures (e.g., absence of public GPs in an area) other may be endogenous and emerge from the individual composition of the neighbourhood (e.g., switching all low and high income individuals to rich and low neighbourhood will also change the neighbourhood context). In general, drawing valid causal inferences in observational epidemiology is difficult and this is especially the case in neighbourhood and health studies (18, 67).

Correspondence between the different measures used to estimate general contextual effects

There is a clear correspondence between the ICC and the AU-ROC so when the ICC is high the AU-ROC is also high. However, the ICC is not influenced by the number of individuals at the neighbourhood since its calculation is based on the neighbourhood variance which, in

turn, is based on differences between neighbourhoods' averages and it is, therefore, standardized for neighbourhood size (i.e., the number of individuals in the neighbourhoods). On the other hand, the AU-ROC is based on the calculation of the TPF and FPF for different thresholds of the predicted probability. Since this predicted probability is an individual level variable, large clusters contribute with more individuals. Because of this difference, it could be possible to find a high ICC but a low AU-ROC if the number of individuals is relatively much larger in some neighbourhood than in others. This situation does not mean that the AU-ROC is a biased measure but, rather, it provides different and useful information. For instance, some large neighbourhoods could have a *high* proportion of individuals visiting a private GP and some small neighbourhoods could have a *low* proportion of individuals visiting a private GP. The ICC would be high indicating that neighbourhoods condition the individual choice of private versus public GP. However, the AU-ROC would be low expressing that most individuals have the same predicted risk, irrespective of whether they visit a private GP or not, and subsequently, that neighbourhoods, in the given context, do not discriminate with accuracy individuals that visit a private GP from those who do not. Otherwise, when neighbourhoods sizes are similar there is a clear correspondence between the ICC and the AU-ROC values (32, 33).

There is also a correspondence between the MOR and the ICC as both are monotone functions of the neighbourhood variance, and this correspondence makes the MOR a measure of general contextual effects. However, the MOR is a measure of probability and not of components of variance as the ICC. The MOR expresses the size of the heterogeneity between the neighbourhoods and the ICC the size of the clustering within neighbourhoods.

The identification of the units of analysis

In contextual epidemiology the individual units are obviously easy to recognize since each individual is delineated by the skin. However, this is not the case when it comes to identifying contextual units. For this purpose, we frequently use geographical and administrative boundaries delineating small areas such as neighbourhoods, blocks, census tracts, or even large territories such as states, counties or countries. We assume that these boundaries condition individual health over and above individual characteristics. Nevertheless, this assumption is rarely validated (3). The components of variance analysis and the use of measures of discriminatory accuracy help us to identify if the definition of neighbourhood we use actually captures a relevant context that influences the health outcome under investigation. Different neighbourhood definitions clearly have different relevance for the same outcome while the same neighbourhood definition may have different relevance for different outcomes.

The "fixed effects approach" for the calculation of the neighbourhood AU-ROC

An alternative to the use of the predicted neighbourhood random effects for the calculation of the AU-ROC is to include the neighbourhoods as fixed-effects dummy variables in a single level logistic regression (i.e., "fixed effects approach"). Using this alternative we obtained an AU-ROC (95% confidence interval) equal to 0.899 (0.891 – 0.899) for visiting a private vs a public GP, and equal to 0.634 (0.628 – 0.640) for use of psychotropic drugs which are very similar to those obtained from Model 2 (general contextual effects) of the multilevel regression analysis. The "fixed effects approach" provides a worthy strategy for a quick evaluation of general contextual effects and it does not require special software for multilevel analyses. However, the fixed effects approach prevents the further study of contextual level variables (e.g., neighbourhood low income). Besides, the model is not parsimonious. For instance in our study we would need to include 217 dummy variables for the 218 neighbourhood's. The fixed effects approach is also susceptible to biased estimation by

random noise if the number of individuals in some neighbourhood's is small, while the prediction of neighbourhood effects in multilevel regression is based on empirical Bayes prediction which protects against this bias by being a so-called shrinkage estimator (12). More fundamentally, the "fixed-effects" logistic model provides inconsistent estimates of the regression coefficients when the number of individuals per neighbourhood is low due to what is known as the *incidental parameter problem* (68) in which case it may be more appropriate to consider conditional logistic regression.

Summary

In observational epidemiology of neighbourhoods and health, there are many unsolved problems concerning the identification of the relevant contexts for specific health outcomes. There are also specific difficulties for drawing causal inferences. Furthermore, in common with other fields in epidemiology, the traditional approach in multilevel analysis of neighbourhood and health maintains a probabilistic approach focused on the analyses of associations and considers the analyses of variance as a secondary task (56). However, some authors, including ourselves (2, 5, 7, 8, 28, 59, 60, 62, 63, 69, 70) stress that the simultaneous consideration of both measures of association and of variance is fundamental in epidemiology (18). The present study clearly illustrates that the bare analysis of measures of association is insufficient for understanding contextual effects on individual health. In fact, naïve interpretations of measures of associations and considering only the statistical "significance" of the neighbourhood variance is misleading and gives an inappropriate base for decision makers. Our study provides concepts and innovative analytical approaches like the use of the AU-ROC that allow improved multilevel analysis of neighbourhood and health.

Finally, performing and interpreting multilevel regression analyses is an interesting task and many technical and conceptual advances have been performed during the last three decades.

However, in the end, the quantitative analysis of contextual influences on individual health may well be unsatisfactory no matter how sophisticated the statistical techniques. Public health would benefit from a stronger humanistic approach that combines multilevel regression and qualitative analyses (71, 72). In any case, epidemiological studies should always provide measures of discriminatory accuracy like the AU-ROC side by side with measures of association.

Acknowledgements

Funding: This work was supported by the Swedish Research Council (PI: Merlo #2013-2484) and by Research founds of the Faculty of Medicine at the Lund University.

References

1. Merlo J. Multilevel analytical approaches in social epidemiology: measures of health variation compared with traditional measures of association. J Epidemiol Community Health. 2003;57(8):550-2.
2. Merlo J, Viciana-Fernandez FJ, Ramiro-Farinas D, Research Group of Longitudinal Database of Andalusian P. Bringing the individual back to small-area variation studies: a multilevel analysis of all-cause mortality in Andalusia, Spain. Soc Sci Med. 2012;75(8):1477-87.
3. Merlo J, Ohlsson H, Lynch KF, Chaix B, Subramanian SV. Individual and collective bodies: using measures of variance and association in contextual epidemiology. J Epidemiol Community Health. 2009;63(12):1043-8.
4. Duncan C, Jones K, Moon G. Do places matter? A multi-level analysis of regional variations in health-related behaviour in Britain. Soc Sci Med. 1993;37(6):725-33.
5. Boyle MH, Willms JD. Place effects for areas defined by administrative boundaries. AmJEpidemiol. 1999;149(6):577-85.
6. Merlo J, Ostergren PO, Hagberg O, Lindstrom M, Lindgren A, Melander A, et al. Diastolic blood pressure and area of residence: multilevel versus ecological analysis of social inequity. J Epidemiol Community Health. 2001;55(11):791-8.
7. Merlo J, Asplund K, Lynch J, Rastam L, Dobson A. Population effects on individual systolic blood pressure: a multilevel analysis of the World Health Organization MONICA Project. Am J Epidemiol. 2004;159(12):1168-79.
8. Clarke P, Wheaton B. Addressing Data Sparseness in Contextual Population Research: Using Cluster Analysis to Create Synthetic Neighborhoods. Sociological Methods Research. 2007;35:311 - 51.
9. Riva M, Gauvin L, Barnett TA. Toward the next generation of research into small area effects on health: a synthesis of multilevel investigations published since July 1998. J Epidemiol Community Health. 2007;61(10):853-61.
10. Merlo J, Chaix B, Ohlsson H, Beckman A, Johnell K, Hjerpe P, et al. A brief conceptual tutorial of multilevel analysis in social epidemiology: using measures of clustering in multilevel logistic regression to investigate contextual phenomena. J Epidemiol Community Health. 2006;60(4):290-7.
11. Merlo J, Chaix B, Yang M, Lynch J, Rastam L. A brief conceptual tutorial on multilevel analysis in social epidemiology: interpreting neighbourhood differences and the effect of neighbourhood characteristics on individual health. J Epidemiol Community Health. 2005;59(12):1022-8.
12. Merlo J, Chaix B, Yang M, Lynch J, Rastam L. A brief conceptual tutorial of multilevel analysis in social epidemiology: linking the statistical concept of clustering to the idea of contextual phenomenon. J Epidemiol Community Health. 2005;59(6):443-9.
13. Merlo J, Yang M, Chaix B, Lynch J, Rastam L. A brief conceptual tutorial on multilevel analysis in social epidemiology: investigating contextual phenomena in different groups of people. J Epidemiol Community Health. 2005;59(9):729-36.
14. Petronis KR, Anthony JC. A different kind of contextual effect: geographical clustering of cocaine incidence in the USA. J Epidemiol Community Health. 2003;57(11):893-900.
15. Rose G. Sick individuals and sick populations. 1985. Bull World Health Organ. 2001;79(10):990-6.
16. Ibanez B, Librero J, Bernal-Delgado E, Peiro S, Lopez-Valcarcel BG, Martinez N, et al. Is there much variation in variation? Revisiting statistics of small area variation in health services research. BMC health services research. 2009;9:60.
17. Sundquist K, Winkleby M, Ahlen H, Johansson SE. Neighborhood socioeconomic environment and incidence of coronary heart disease: a follow-up study of 25,319 women and men in Sweden. Am J Epidemiol. 2004;159(7):655-62.

18. Merlo J. Invited commentary: multilevel analysis of individual heterogeneity-a fundamental critique of the current probabilistic risk factor epidemiology. American Journal of Epidemiology. 2014;180(2):208-12.
19. Pepe MS, Janes H, Longton G, Leisenring W, Newcomb P. Limitations of the odds ratio in gauging the performance of a diagnostic, prognostic, or screening marker. Am J Epidemiol. 2004;159(9):882-90.
20. Pencina MJ, D'Agostino RB, Pencina KM, Janssens AC, Greenland P. Interpreting incremental value of markers added to risk prediction models. Am J Epidemiol. 2012;176(6):473-81.
21. Pencina MJ, D'Agostino RB, Sr., Steyerberg EW. Extensions of net reclassification improvement calculations to measure usefulness of new biomarkers. Stat Med. 2011;30(1):11-21.
22. Pencina MJ, D'Agostino RB, Sr., D'Agostino RB, Jr., Vasan RS. Evaluating the added predictive ability of a new marker: From area under the ROC curve to reclassification and beyond. Statistics in Medicine. 2008;27(2):157-72.
23. Pepe MS. Problems with risk reclassification methods for evaluating prediction models. Am J Epidemiol. 2011;173(11):1327-35.
24. Hidden J, Gerds T. Evaluating the impact of novel biomarkers: Do not rely on IDI and NRI. Department of Biostatistics, University of Copenhagen, Denmark. 2012;Research Report 12/08.
25. Goldstein H, Browne W, Rasbash J. Partitioning variation in generalised linear multilevel models. Understanding Statistics. 2002;1:223-32.
26. Browne WJ, Subramanian SV, Jones K, Goldstein H. Variance partitioning in multilevel logistic models that exhibit overdispersion. Journal of the Royal Statistical Society: Series A (Statistics in Society). 2005;168(3):599-613.
27. Li J, Gray BR, Bates DM. An Empirical Study of Statistical Properties of Variance Partition Coefficients for Multi-Level Logistic Regression Models. Communications in Statistics - Simulation and Computation. 2008;37:2010–26.
28. Larsen K, Merlo J. Appropriate assessment of neighborhood effects on individual health: integrating random and fixed effects in multilevel logistic regression. Am J Epidemiol. 2005;161(1):81-8.
29. Larsen K, Petersen JH, Budtz-Jorgensen E, Endahl L. Interpreting parameters in the logistic regression model with random effects. Biometrics. 2000;56(3):909-14.
30. Bennett B. Use of the tetrachoric correlation model in assessing clinical tests. Biometrical Journal. 1980;22(4):335–8.
31. Lorenzo-Seva U, Ferrando PJ. TETRA-COM: a comprehensive SPSS program for estimating the tetrachoric correlation. Behavior research methods. 2012;44(4):1191-6.
32. Wagner P, Merlo J. Measures of discriminatory accuracy in multilevel analysis. European journal of epidemiology. 2013;28(1, Supplement):135.
33. Wagner P, Merlo J. Discriminatory accuracy of a random effect in multilevel logistic regression. 20th IEA World Congress of Epidemiology (WCE2014). 2014.
34. Wettermark B, Hammar N, Fored CM, Leimanis A, Otterblad Olausson P, Bergman U, et al. The new Swedish Prescribed Drug Register--opportunities for pharmacoepidemiological research and experience from the first six months. Pharmacoepidemiology and drug safety. 2007;16(7):726-35.
35. WHO Collaborating Centre for Drug Statistics Methodology. ATC/DDD Index. Available from: http://wwwwhoccno/atc_ddd_index/. 2013.
36. Sweden S. Product catalogue. Statistics Sweden's Market Profiles. 2014;http://www.scb.se/marketprofiles/.
37. Leckie G, Goldstein H. Understanding Uncertainty in School League Tables. Fiscal Studies. 2011;DOI: 10.1111/j.1475-5890.2011.00133.
38. Leckie G, Goldstein H. The limitations of using school league tables to inform school choice. Journal of the Royal Statistical Society: Series A (Statistics in Society), . 2009(172):835-51.

39. Leckie G, Goldstein H. A note on "The limitations of using school league tables to inform school choice". Journal of the Royal Statistical Society: Series A (Statistics in Society), . 2011(174):833-6.
40. Browne WJ. MCMC Estimation in MLwiN (Version 2.13) Centre for Multilevel Modelling, University of Bristol http://wwwbristolacuk/cmm/software/mlwin/download/mcmc-printpdf. 2009;Downloaded, 27 Mars 2014.
41. Spiegelhalter DJ, Best N, Carlin BP, Linde AVD. Bayesian measures of model complexity and fit. Journal of the Royal Statistical Society Series C, Applied statistics. 2002;64:583-639.
42. Lunn D, Jackson C, Best N, Thomas A, Spiegelhalter D. The BUGS book: A practical introduction to Bayesian analysis. . Boca Raton, FL: Chapman & Hall/CRC Texts in Statistical Science. 2012.
43. Yang M, Eldridge S, Merlo J. Multilevel survival analysis of health inequalities in life expectancy. International journal for equity in health. 2009;8:31.
44. Merlo J, Ohlsson H, Chaix B, Lichtenstein P, Kawachi I, Subramanian SV. Revisiting causal neighborhood effects on individual ischemic heart disease risk: a quasi-experimental multilevel analysis among Swedish siblings. Soc Sci Med. 2013;76(1):39-46.
45. Mulinari S, Juárez S, Wagner P, Merlo J. Does maternal country of birth matter for understanding offspring's birthweight? A multilevel analysis of individual heterogeneity in Sweden. . PLOS-ONE (in press). 2015.
46. Maas CJM, Hox JJ. The influence of violations of assumptions on multilevel parameter etimates and their standar errors. Computational statistics & data analysis. 2004;46:427-40.
47. Goldstein H, Noden P. Modelling social segregation. Oxford Review of Education,. 2003;29(2):225-37.
48. Leckie G, Goldstein H. A multilevel modelling approach to measuring changing patterns of ethnic composition and segregation among London secondary schools, 2001-2010. Journal of the Royal Statistical Society: Series A (Statistics in Society), . 2015;178:405-24.
49. Leckie G, Pillinger R, Jones K, Goldstein H. Multilevel Modeling of Social Segregation. JOURNAL OF EDUCATIONAL AND BEHAVIORAL STATISTICS 2012;37 (1):3-30.
50. Johnell K, Merlo J, Lynch J, Blennow G. Neighbourhood social participation and women's use of anxiolytic-hypnotic drugs: a multilevel analysis. J Epidemiol Community Health. 2004;58(1):59-64.
51. Collaboration. Farmakoterapi vid ångest – Behandlingsrekommendation. Information från Läkemedelsverket 4:2006, Sweden. 2006.
52. Hälso- och sjukvårdslag. 1982:763. http://www.riksdagen.se/webbnav/index.aspx?nid=3911&bet=1982:763, (1982).
53. Beckman A. Country of birth and socioeconomic disparities in utilisation of health care and disability pension – a multilevel approach. [Thesis doctoral]. Malmö Lunds University; 2005.
54. Veblen T. The Theory of the Leisure Class. Baltimore: Penguin Books; 1994.
55. Pickett KE, Pearl M. Multilevel analyses of neighbourhood socioeconomic context and health outcomes: a critical review. JEpidemiolCommunity Health. 2001;55(2):111-22.
56. Diez Roux AV. Next steps in understanding the multilevel determinants of health. J Epidemiol Community Health 1470-2738 (Electronic). 2008;62(11):957-9.
57. Merlo J, Wagner P. The tyranny of the averages and the indiscriminate use of risk factors in public health: a call for revolution. European journal of epidemiology. 2013;28(1, Supplement):148.
58. Mitchell R. Multilevel modeling might not be the answer. Environ Plan A. 2001;33:1357-60.
59. Rasbash J, Leckie G, Pillinger R, Jenkins J. Children's educational progress: partitioning family, school and area effects. Journal of the Royal Statistical Society: Series A (Statistics in Society). 2010;173(3):657-82.
60. Leckie G. The complexity of school and neighbourhood effects and movements of pupils on school differences in models of educational achievement. Journal of the Royal Statistical Society: Series A (Statistics in Society). 2009;172(537-554).

61. Morenoff JD. Neighborhood mechanisms and the spatial dynamics of birth weight. AJS. 2003;108(5):976-1017.
62. Ohlsson H, Merlo J. Place effects for areas defined by administrative boundaries: a life course analysis of mortality and cause specific morbidity in Scania, Sweden. Soc Sci Med. 2011;73(8):1145-51.
63. Sariaslan A, Langstrom N, D'Onofrio B, Hallqvist J, Franck J, Lichtenstein P. The impact of neighbourhood deprivation on adolescent violent criminality and substance misuse: a longitudinal, quasi-experimental study of the total Swedish population. International Journal of Epidemiology. 2013;42(4):1057-66.
64. Leckie G. Cross-classified Multilevel Models - Concepts. LEMMA VLE Module 12, 1-60. . 2013.
65. Leckie G. Multiple Membership Multilevel Models - Concepts. LEMMA VLE Module 13, 1-61. 2013.
66. Browne WJ, Goldstein H, Rasbash J. Multiple membership multiple classification (MMMC) models. Statistical Modelling. 2001;1(2):103-24.
67. Oakes JM. The (mis)estimation of neighborhood effects: causal inference for a practicable social epidemiology. Soc Sci Med. 2004;58(10):1929-52.
68. Neyman J, Scott E L. Consistent estimates based on partially consistent observations. Econometrica 16, 1–32. Econometrica 1948;16:1-32.
69. Dundas R, Leyland AL, Macintyre S. Early life school, neighbourhood and family influences on adult health: a multilevel cross-classified analysis of the Aberdeen Children of the 1950s study. American Journal of Epidemiology. 2014;AJE-00606-2013.R5.
70. Leyland AH, Naess O. The effect of area of residence over the life course on subsequent mortality. Journal of the Royal Statistical Society Series A. 2009;172(3):555-78.
71. Cummins S, Curtis S, Diez-Roux AV, Macintyre S. Understanding and representing 'place' in health research: A relational approach. Soc Sci Med. 2007;65(9):1825-38.
72. Macintyre S, Ellaway A, Cummins S. Place effects on health: how can we conceptualise, operationalise and measure them? Soc Sci Med. 2002;55(1):125-39.

Figure
Click here to download Figure: Fig. 1.TIF

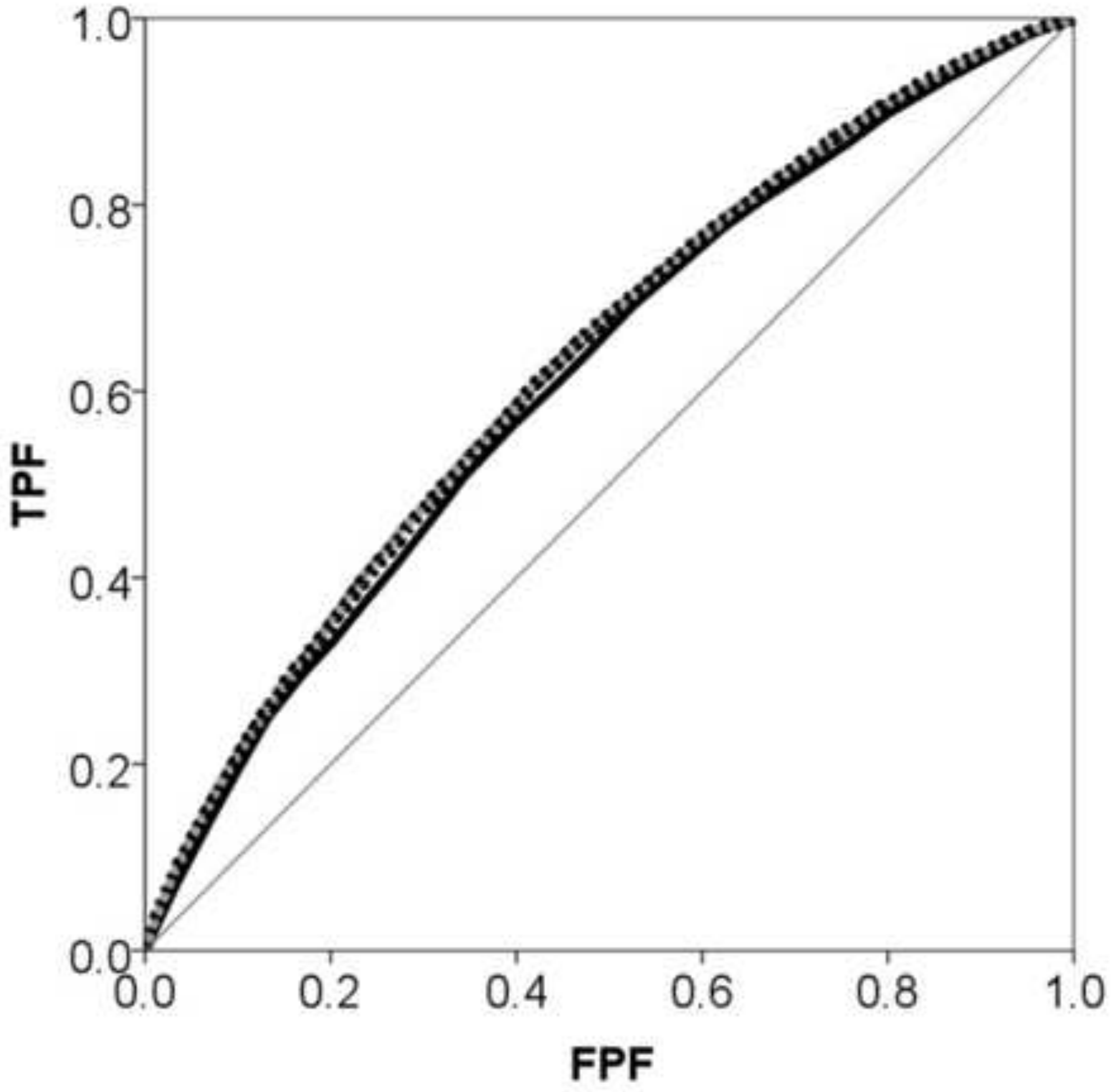

Figure 1

Figure
Click here to download Figure: Fig. 2.TIF

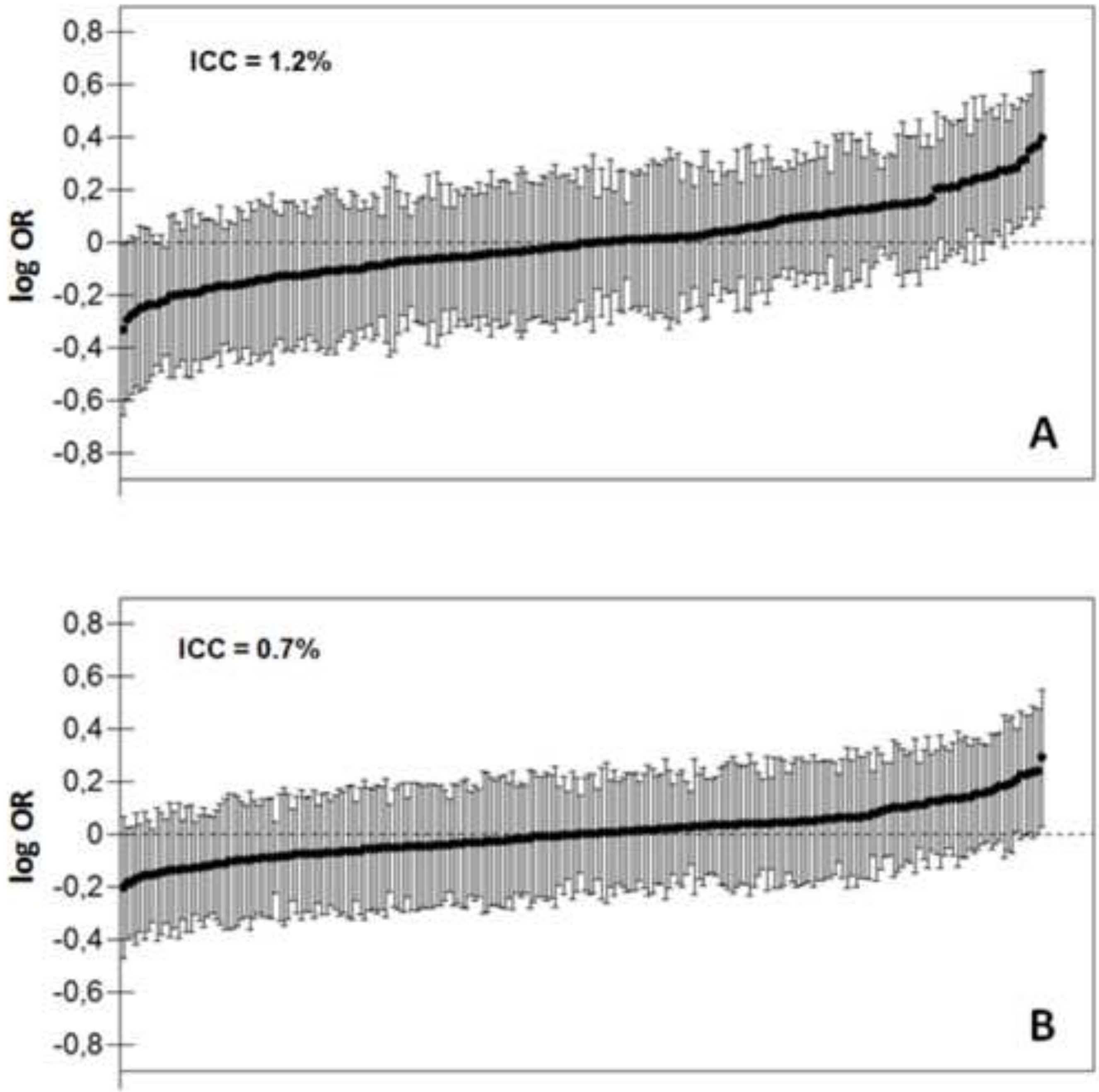

Figure 2

Figure
Click here to download Figure: Fig. 3.TIF

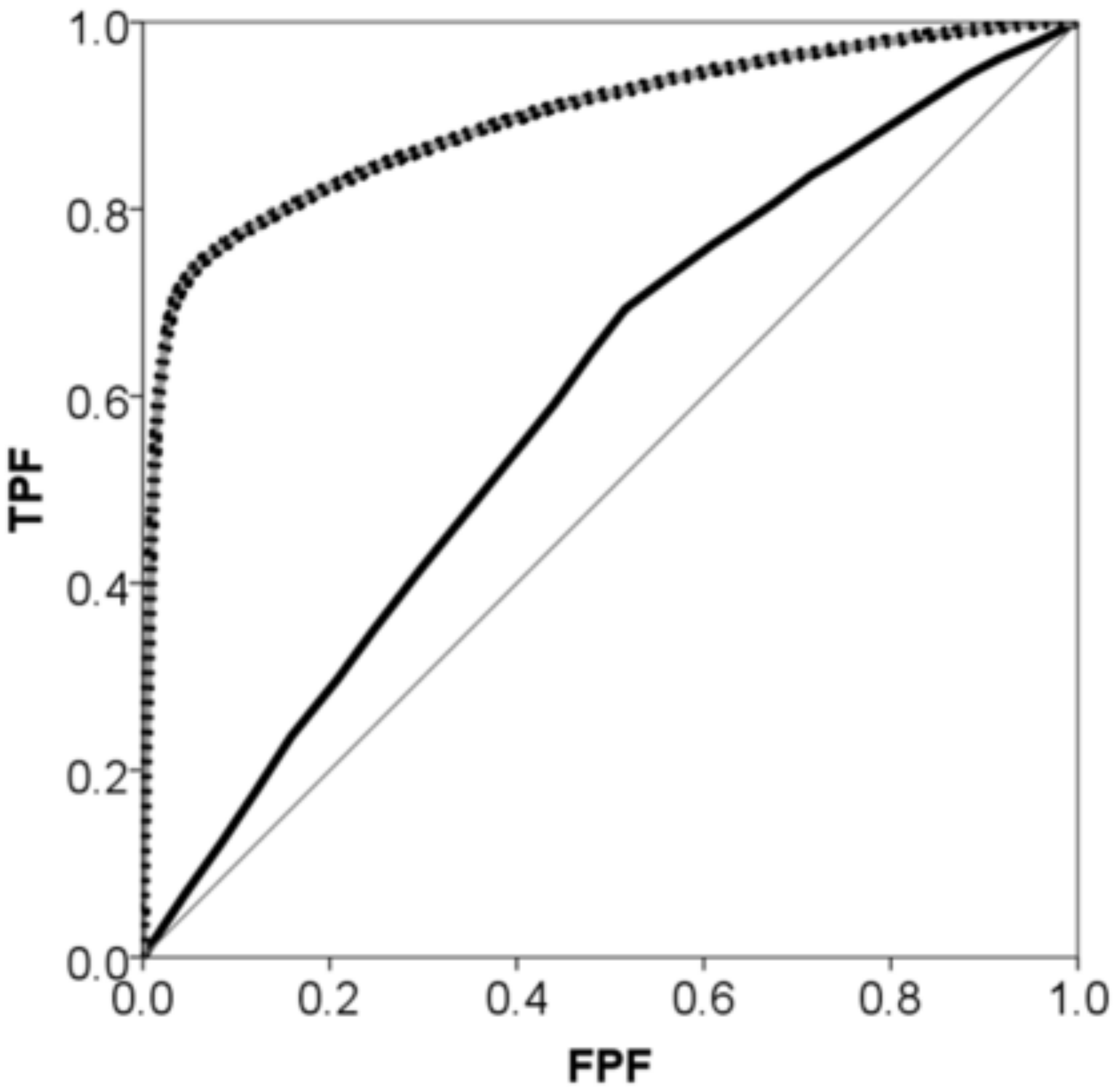

Figure 3

Figure
Click here to download Figure: Fig. 4.TIF

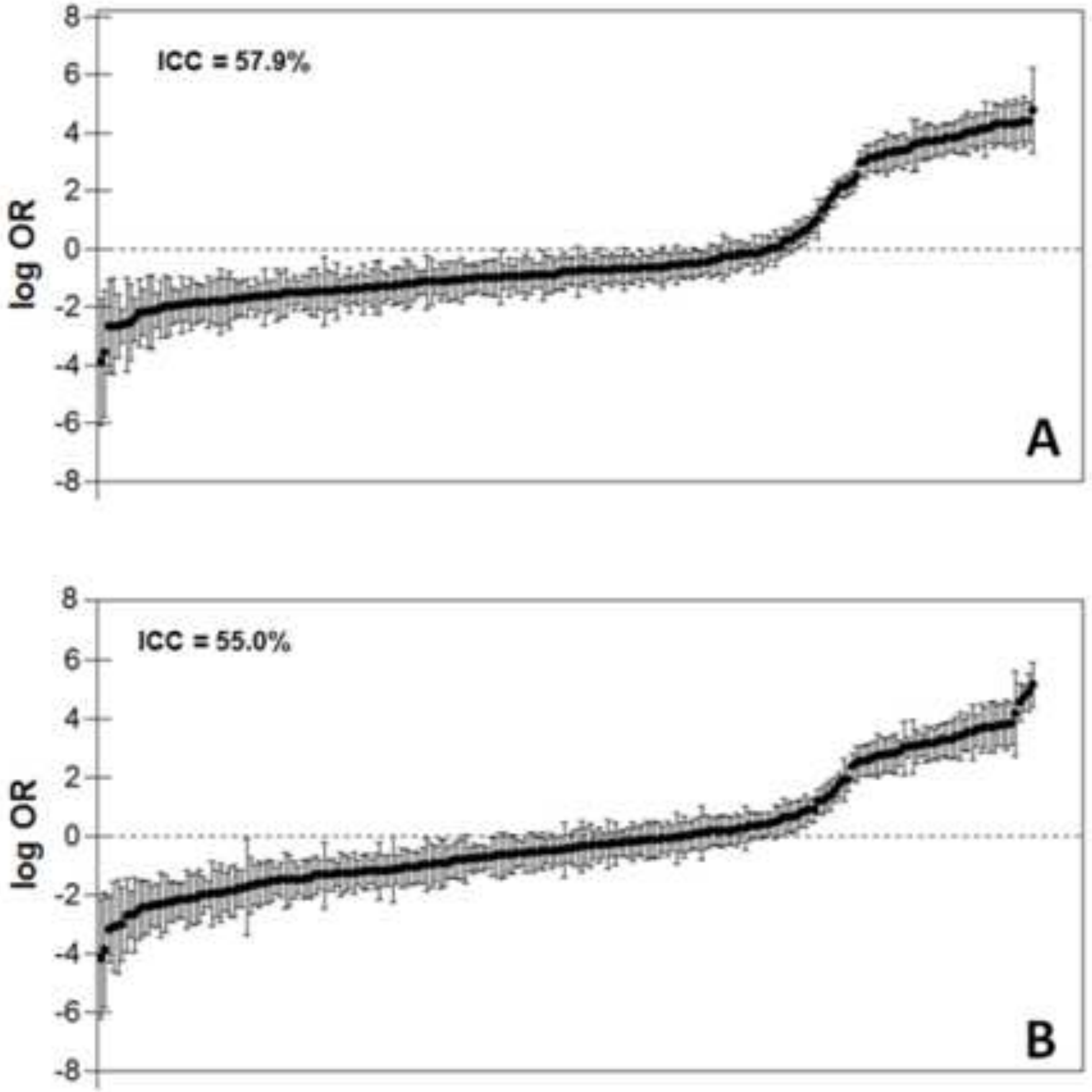

Figure 4

Click here to download Supporting Information: stata.do

Click here to download Supporting Information: data_43291_Stata.dta

Click here to download Supporting Information: data_43291_Excel.xlsx

Click here to download Supporting Information: data_43291_spss.sav

Click here to download Supporting Information: data_43291_MLwiN.wsz

Click here to download Supporting Information: GP__m1.wsz

Click here to download Supporting Information: GP__m2.wsz

Click here to download Supporting Information: GP__m3.wsz

Click here to download Supporting Information: psy__m1.wsz

Click here to download Supporting Information: psy__m2.wsz

Click here to download Supporting Information: psy__m3.wsz

Supporting Information
Click here to download Supporting Information: MOR-IOR-POOR-ICC-PCV.xls
