# Supplementary material for: Does the Neighborhood Area of Residence Influence Non-Attendance in an Urban Mammography Screening Program? A Multilevel Study in a Swedish City
Source: PLoS One. 2015 Oct 13;10(10):e0140244. doi: 10.1371/journal.pone.0140244 (PMC4604149; doi:10.1371/journal.pone.0140244)
Supplement: S1 Table — Multivariable logistic regression modeling screening related variables and women’s individual sociodemographic characteristics and the odds of mammography non-attendance (n = 29,901). (DOCX) [file pone.0140244.s002.docx]

**S1 Table. Coefficients used for women’s individual risk score.**

Multivariable logistic regression modelling screening related variables and women’s individual sociodemographic characteristics and the odds of mammography non-attendance (n=29,901).

|  | **ß-coefficient** | **P-value** | **OR (95% CI)** |
| --- | --- | --- | --- |
| Number of invitations | -0.132 | <0.001 | 0.88 (0.86-0.89) |
| Year |  |  |  |
| 2005 | 1.537 | <0.001 | 4.65 (4.04-5.36) |
| 2006 | 1.391 | <0.001 | 4.02 (3.57-4.53) |
| 2007 | 0.076 | 0.095 | 1.08 (0.99-1.17) |
| 2008 | -0.254 | <0.001 | 0.78 (0.71-0.85) |
| 2009 |  |  | Reference |
| Season |  |  |  |
| Fall |  |  | Reference |
| Winter | 0.368 | <0.001 | 1.45 (1.33-1.57) |
| Spring | 0.394 | <0.001 | 1.48 (1.36-1.61) |
| Summer | 0.254 | <0.001 | 1.30 (1.16-1.46) |
| Age group |  |  |  |
| 48-50 | 1.065 | <0.001 | 2.90 (2.39-3.52) |
| 51-55 | 1.082 | <0.001 | 2.95 (2.50-3.48) |
| 56-60 | 0.754 | <0.001 | 2.13 (1.83-2.48) |
| 61-65 | 0.813 | <0.001 | 2.25 (1.94-2.61) |
| 66-70 | 0.502 | <0.001 | 1.65 (1.41-1.93) |
| 71-75 |  |  | Reference |
| Marital status |  |  |  |
| Married |  |  | Reference |
| Widow | 0.476 | <0.001 | 1.61 (1.40-1.85) |
| Divorced | 0.619 | <0.001 | 1.86 (1.72-2.01) |
| Never married | 0.603 | <0.001 | 1.83 (1.66-2.02) |
| Number of children |  |  |  |
| 0 |  |  | Reference |
| 1 | -0.046 | 0.304 | 0.96 (0.88-1.04) |
| >1 | -0.133 | 0.012 | 0.88 (0.79-0.97) |
| Education (years) |  |  |  |
| >12 |  |  | Reference |
| 10-12 | 0.172 | 0.721 | 1.01 (0.94-1.10) |
| <=9 | 0.619 | <0.001 | 1.18 (1.08-1.30) |
| Missing | 0.579 | <0.001 | 1.78 (1.43-2.22) |
| Income (100 SEK) |  |  |  |
| High (≥1697) |  |  | Reference |
| Middle-High (1233-1696) | 0.051 | 0.317 | 1.05 (0.95-1.16) |
| Middle-Low (924-1232) | 0.210 | <0.001 | 1.23 (1.12-1.36) |
| Low (<923) | 0.326 | <0.001 | 1.39 (1.24-1.54) |
| Not employed | 0.618 | <0.001 | 1.86 (1.71-2.01) |
| Country of birth |  |  |  |
| Sweden |  |  | Reference |
| Nordic | 0.117 | 0.307 | 1.12 (0.90-1.41) |
| Europe | 0.429 | <0.001 | 1.54 (1.40-1.68) |
| Other | 0.455 | <0.001 | 1.58 (1.39-1.79) |
| Country of citizenship |  |  |  |
| Sweden |  |  | Reference |
| Nordic | -0.009 | 0.955 | 0.99 (0.74-1.33) |
| Europe | 0.298 | 0.001 | 1.35 (1.13-1.60) |
| Other | 0.157 | 0.196 | 1.17 (0.92-1.48) |
| Time in Sweden (years) |  |  |  |
| >5 |  |  | Reference |
| <=5 | -0.089 | 0.572 | 0.91 (0.67-1.25) |
| Missing | 0.112 | 0.597 | 1.12 (0.74-1.69) |
| Migration across Swedish boarder more than once | 0.368 | <0.001 | 1.44 (1.26-1.65) |
|  |  |  |  |
